# Intestinal epithelial *N*-acylphosphatidylethanolamine phospholipase D links dietary fat to metabolic adaptations in obesity and steatosis

**DOI:** 10.1038/s41467-018-08051-7

**Published:** 2019-01-28

**Authors:** Amandine Everard, Hubert Plovier, Marialetizia Rastelli, Matthias Van Hul, Alice de Wouters d’Oplinter, Lucie Geurts, Céline Druart, Sylvie Robine, Nathalie M. Delzenne, Giulio G. Muccioli, Willem M. de Vos, Serge Luquet, Nicolas Flamand, Vincenzo Di Marzo, Patrice D. Cani

**Affiliations:** 1 Metabolism and Nutrition Research Group, Louvain Drug Research Institute, Walloon Excellence in Life sciences and BIOtechnology (WELBIO), UCLouvain, Université catholique de Louvain, Av. E. Mounier, 73 B1.73.11, 1200 Bruxelles, Belgium; 2 Institut Curie, CNRS, Unité 144, 75248 Paris, France; 3 Bioanalysis and Pharmacology of Bioactive Lipids Research Group, Louvain Drug Research Institute, UCLouvain, Université catholique de Louvain, 1200 Bruxelles, Belgium; 4 Laboratory of Microbiology, Wageningen University, 6708WE Wageningen, the Netherlands; 5 Université Paris Diderot, Sorbonne Paris Cité, BFA, UMR8251, CNRS, 75013 Paris, France; 6 Quebec Heart and Lung Institute Research Centre, Université Laval, G1V 0A6 Quebec City, Canada; 7 Institute of Nutrition and Functional Foods, Université Laval, G1V 0A6 Quebec City, Canada; 8 Endocannabinoid Research Group, Institute of Biomolecular Chemistry, Consiglio Nazionale delle Ricerche, 80078 Pozzuoli, Napoli, Italy

## Abstract

Variations in *N*-acylethanolamines (NAE) levels are associated with obesity and metabolic comorbidities. Their role in the gut remains unclear. Therefore, we generated a mouse model of inducible intestinal epithelial cell (IEC)-specific deletion of *N*-acylphosphatidylethanolamine phospholipase D (NAPE-PLD), a key enzyme involved in NAE biosynthesis (*Napepld*^∆IEC^). We discovered that *Napepld*^∆IEC^ mice are hyperphagic upon first high-fat diet (HFD) exposure, and develop exacerbated obesity and steatosis. These mice display hypothalamic *Pomc* neurons dysfunctions and alterations in intestinal and plasma NAE and 2-acylglycerols. After long-term HFD, *Napepld*^∆IEC^ mice present reduced energy expenditure. The increased steatosis is associated with higher gut and liver lipid absorption. *Napepld*^∆IEC^ mice display altered gut microbiota. *Akkermansia muciniphila* administration partly counteracts the IEC NAPE-PLD deletion effects. In conclusion, intestinal NAPE-PLD is a key sensor in nutritional adaptation to fat intake, gut-to-brain axis and energy homeostasis and thereby constitutes a novel target to tackle obesity and related disorders.

## Abstract (web summary)

Obesity is associated with altered *N*-acylethanolamine levels (NAE). Here the authors show that deletion of the gene encoding *N*-acylphosphatidylethanolamine phospholipase D, a key enzyme for NAE synthesis, in intestinal cells of mice leads to the development of obesity and hepatic steatosis via a mechanism involving the gut-brain axis.

## Introduction

Obesity is a pandemic affecting more than 10% of the population worldwide^1^. Besides increased adipose tissue mass, obesity is associated with chronic inflammation and alterations in nearly all tissues of metabolic relevance. This predisposes obese individuals to manifestations of the metabolic syndrome such as insulin resistance, hepatic steatosis and cardiovascular disorders. However, the mechanisms linking obesity and such metabolic alterations remain poorly understood. It is crucial to deepen our understanding of the pathophysiology of obesity and its comorbidities.

The endocannabinoid system (ECS) consists of ubiquitous bioactive lipids regulating glucose and lipid metabolism, food intake, and inflammation through various receptors^2^. One of the best characterized endocannabinoids, the *N*-acylethanolamine (NAE) anandamide (*N*-arachidonoylethanolamine, AEA) stimulates the canonical cannabinoid receptors CB1 and CB2^3^. Other NAE such as oleoylethanolamide (OEA), stearoylethanolamide (SEA), linoleoylethanolamide (LEA) and palmitoylethanolamide (PEA) are structurally close to and share biosynthetic pathways with AEA but do not activate CB1 or CB2. They are considered endocannabinoid-like molecules acting on other receptors such as PPARα or GPR119^4,5^. Levels of endocannabinoids and related mediators are directly regulated by the activity of their synthesizing and degrading enzymes. NAE are mainly synthesized by NAPE-PLD, although studies in *Napepld*-knockout (KO) mice have described alternative pathways for AEA synthesis^6^. NAE degradation is mediated by the activity of the fatty acid amide hydrolase (FAAH) and NAE-hydrolyzing acid amidase (NAAA)^3^.

Obesity is associated with altered NAE levels and excessive CB1 activation leading to lipid accumulation and inflammation in the liver, muscles and adipose tissue, and impaired glucose tolerance^2^. Consistently, *Cnr1* (encoding CB1)-KO mice are protected against diet-induced obesity. Enzymes of the ECS also play a role in the development of metabolic syndrome, as *Faah*-KO mice become obese and insulin resistant even on a control diet^7^. Interestingly, single nucleotide polymorphisms in *Napepld* have been correlated to obesity in humans^8^. However, the influence of this enzyme is less clear as whole-body *Napepld*-KO mice do not display changes in body composition or glucose metabolism^9^. Of note, peripheral NAE levels remain unaffected in whole-body *Napepld*-KO mice^10^. Conversely, we recently showed that adipocyte-specific *Napepld* deletion decreases adipose tissue NAE levels and favors obesity development in control diet-fed mice^11^. It is therefore of interest to study the role of NAPE-PLD in specific tissues of relevance in obesity.

The intestinal epithelium regulates energy metabolism through its roles in nutrient absorption and via the various hormones secreted by enteroendocrine cells (EEC)^12^. It is also a major source of endocannabinoids and related compounds modulating food intake^13–16^. Short-term fat exposure in the stomach induces jejunal AEA mobilization, while duodenal fat exposure leads to OEA synthesis, contributing to the fine-tuning of dietary fat intake^17,18^. Furthermore, recent data highlighted the importance of intestinal ECS in the regulation of lipid absorption, enteroendocrine secretions and the gut barrier function^3,5^. Intestinal NAE levels are decreased during diet-induced obesity^19–21^. Whether these changes play a role in the development of the metabolic syndrome remains to be investigated.

To assess the importance of intestinal NAE in obesity, we generated a model of inducible *Napepld* deletion specifically in intestinal epithelial cells (*Napepld*^∆IEC^ mice) and studied its consequences on metabolism in physiological conditions and during diet-induced obesity. HFD-fed *Napepld*^∆IEC^ mice are more sensitive to obesity and steatosis together with changes in food intake, altered hypothalamic regulation of energy homeostasis, lower energy expenditure and modified gut microbiota. As we previously demonstrated that *Akkermansia muciniphila*, a gut microbe with beneficial effects during obesity, could exert its effects through modulation of intestinal bioactive lipids related to the ECS, we tested whether intestinal NAPE-PLD mediated these effects. *Napepld*^∆IEC^ mice remain sensitive to protective effects of *A. muciniphila*, suggesting that intestinal NAPE-PLD is not required for the beneficial effects of this bacterium against the metabolic syndrome. Our results highlight additional roles of intestinal endocannabinoids and related mediators in the regulation of food intake through the gut-brain axis and support their importance in the gut-liver axis. They also highlight new mechanisms of interactions between *A. muciniphila* and its host in the context of obesity.

## Results

### Validation of the *Napepld*^∆IEC^ mouse model

**Fig. 1:**
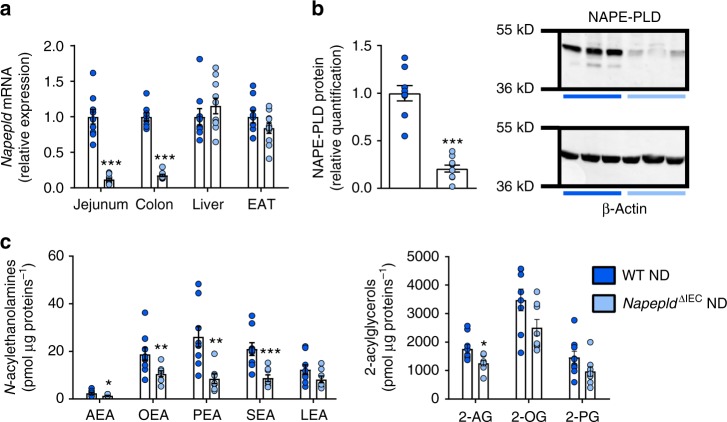
Validation of IEC *Napepld* deletion. **a** *Napepld* mRNA expression in the jejunum, colon, liver and epididymal adipose tissue (EAT) in ND-fed WT and *Napepld*^∆IEC^ mice (*n* = 8–10). **b** NAPE-PLD protein levels in the colon. Representative western-blot of NAPE-PLD and β-Actin (*n* = 9–10). **c** Levels of *N*-acylethanolamines and 2-acylglycerols in small intestinal epithelial cells of ND-fed WT and *Napepld*^∆IEC^ mice (*n* = 7–9) were determined by using high-performance liquid chromatography-MS using an LTQ Orbitrap mass spectrometer as described in the methods. Dark blue: WT ND mice, light blue: *Napepld*^∆IEC^ ND mice. Data are presented as the mean ± s.e.m. *, ** and *** indicate a significant difference versus WT ND (Respectively *P* < 0.05, *P* < 0.01 and *P* < 0.001) according to Mann–Whitney test

Villin-Cre^ERT2^ mice^22^ were crossed with *Napepld*^*l*ox/lox^ mice^11^ to generate *Napepld*^∆IEC^ mice. To validate the model, we quantified *Napepld* gene expression in multiple tissues of mice fed a control diet (ND). Gene expression was reduced in the jejunum and colon of *Napepld*^∆IEC^ mice (Fig. 1a). No reduction was observed in the liver or epididymal adipose tissue (EAT), supporting the specificity of the deletion in intestinal segments (Fig. 1a). The deletion was confirmed by western blot analysis; *Napepld*^∆IEC^ mice had 5 times lower protein levels of NAPE-PLD than wild-type (WT) mice (Fig. 1b).

Finally, we quantified endocannabinoids (eCB) and eCB-like (i.e., NAE and monoacylglycerols) compounds in intestinal epithelial cells isolated from the small intestine of WT and *Napepld*^∆IEC^ mice. All NAE levels were affected by the deletion, with significant decreases in AEA (42%), OEA (44%), PEA (68%), SEA (58%) and a trend for LEA (33%). Conversely, only 2-arachidonoylglycerol was decreased, while other acylglycerols were not significantly affected (Fig. 1c).

### *Napepld*^∆IEC mice^ are hyperphagic upon first exposure to HFD

Duodenal lipid infusions stimulate intestinal OEA production, leading to increased satiety upon fat exposure^17^, whereas small-intestinal OEA production is disrupted during chronic fat feeding^21^. However, the origin of this defect has not been identified. As *Napepld*^∆IEC^ mice display lower intestinal NAE levels, we reasoned that their food intake could be modified directly after a first high-fat diet (HFD) exposure thereby providing a first proof of concept that NAPE-PLD *per se* can play a major role in this setting.

**Fig. 2:**
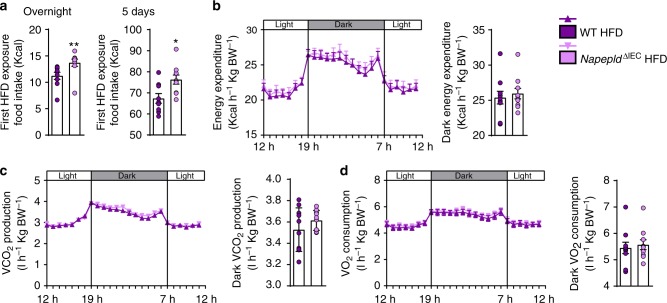
*Napepld*^∆IEC^ are hyperphagic upon early HFD exposure without changes in energy expenditure. **a** HFD intake (Kcal) after 24 h and 5 days of feeding (*n* = 9–10). **b** Light and dark cycle energy expenditure (Kcal h^−1^ Kg body weight^−1^) measured in metabolic chambers during indirect calorimetry studies. **c** Light and dark cycle of CO_2_ production (l h^−1^ Kg body weight^−1^). **d** Light and dark cycle of O_2_ consumption (l h^−1^ kg body weight^−1^). Purple: WT HFD mice and pink: *Napepld*^∆IEC^ HFD mice. Data are presented as the mean ± s.e.m. * and ** indicate a significant difference versus WT HFD (Respectively *P* < 0.05 and *P* < 0.01) according to Mann–Whitney test

Therefore, we recorded individual HFD intake of WT and *Napepld*^∆IEC^ mice following their first exposure to the diet using metabolic cages. Consistent with our hypothesis, *Napepld*^∆IEC^ mice ate significantly more during the first overnight exposure to the HFD and the difference was maintained after 5 days (Fig. 2a). As intestinal NAE can also affect energy metabolism, we measured energy expenditure during the same period. Energy expenditure, CO_2_ production, and O_2_ consumption were similar in both groups (Fig. 2b–d).

Intestinal NAE can act through the gut-brain axis to affect gut hormones but also the central regulation of food intake by acting first on the arcuate nucleus (ARC) and eventually in the paraventricular nucleus (PVN).

**Fig. 3:**
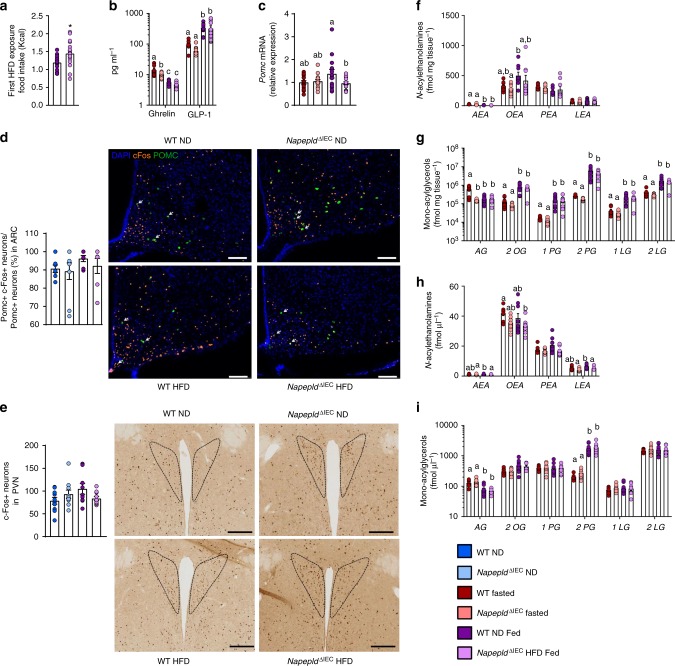
Hyperphagia in Napepld^*∆IEC*^ mice is linked to alterations in Pomc neurons, NAE, and mono-acylglycerols. **a** HFD intake (Kcal) (*n* = 17). **b** Ghrelin and GLP-1 levels (pg ml^-1^) in the portal vein (*n* = 7–9). **c** Relative mRNA expression of *Pomc* in the hypothalamus (*n* = 15–17). **d** Percentage of Pomc and c-Fos positive neurons among Pomc positive neurons in the ARC (*n* = 7–10) and representative images showing double IF-TSA staining in the ARC (bregma −1,70 mm) (scale bar = 100 μm), magnification 20×. White arrows indicate activated neurons (c-Fos positive, in orange) expressing POMC (in green). **e** c-Fos positive neurons in the PVN (*n* = 9–10) and representative bright field images of c-Fos immunohistochemistry in the PVN (bregma −0,58 mm) (scale bar = 200 µm), magnification 10×, black dashed line is delimiting the PVN. **f** *N*-acylethanolamines (fmol mg^−1^) measured in the jejunum (*n* = 7–9) using Shimadzu 8050 triple quadrupole mass spectrometer. **g** mono-acylglycerols (AG = 1AG + 2AG) (fmol mg^−1^) measured in the jejunum (*n* = 7–9). **h** *N*-acylethanolamines (fmol μl^−1^) measured in the portal vein (*n* = 7–9). **i** Mono-acylglycerols (AG = 1AG + 2AG) (fmol μl^−1^) measured in the portal vein (*n* = 7–9) using Shimadzu 8050 triple quadrupole mass spectrometer. **a**–**c** and **g**–**j** are measured in WT and *Napepld*^*∆IEC*^ mice either fasted or after 4 h of HFD intake, **d**–**f** are measured in WT and *Napepld*^*∆IEC*^ mice after 1 h of ND or HFD intake. Data in **a** and **c** correspond to the results of two independent experiments. Dark blue: WT ND mice, light blue: *Napepld*^∆IEC^ ND mice, red: WT fasted mice, salmon: *Napepld*^∆IEC^ fasted mice, purple: WT HFD mice and pink: *Napepld*^∆IEC^ HFD mice. Data are presented as the mean ± s.e.m. Asterisk (*) indicates a significant difference versus WT HFD (*P* < 0.05) according to Mann–Whitney test. Data with different superscript letters are significantly different (*P* < 0.05) according to regular two-way ANOVA followed by Tukey’s post-hoc test

*Napepld*^∆IEC^ mice ingest significantly more food during the 4 h of first HFD exposure compared to WT mice (Fig. 3a). We therefore quantified levels of key gut hormones modulating food intake. Expectedly, HFD exposure significantly decreased portal ghrelin and increased portal GLP-1 levels as compared to fasted mice (Fig. 3b). However, *Napepld*^∆IEC^ and WT mice exhibited similar profiles under HFD. These results suggest that these gut hormones do not contribute to the phenotype observed in *Napepld*^∆IEC^ mice.

The final integration of signals regulating food intake occurs in the hypothalamus. To dissect the potential mechanisms explaining the modification of energy intake, we investigated hypothalamic expression of anorectic (i.e., *Pomc*, encoding pro-opiomelanocortin and *Cart*, encoding cocaine-related and amphetamine-related transcript) and orexigenic (i.e., *Npy*, encoding Neuropeptide Y and *Agrp*, encoding Agouti-related peptide) peptides known to be modulated early after HFD feeding^23^.

Interestingly, HFD feeding was sufficient to induce a significant increase in *Pomc* mRNA expression in WT animals compared to fasted animals, whereas fed *Napepld*^∆IEC^ mice exhibited similar expression levels to those of fasted mice (Fig. 3c). Intestinal AEA and OEA can act through the gut-brain axis to affect the central regulation of food intake notably by regulating hypothalamic *Oxytocin* expression^4,15,24^. Remarkably, neither the expression of *Oxytocin*, nor that of *Cart*, *Npy*, and *Agrp* were affected (Supplementary Figure 2).

Additionally, we quantified c-Fos positive neurons, a canonical marker of neuron activation. After an overnight fasting, we exposed WT and *Napepld*^∆IEC^ mice to ND or HFD and quantified c-Fos in the brain. Activation of hypothalamic POMC neurons tends to be higher in HFD-fed WT mice as compared to ND-fed WT mice (Fig. 3d), whereas this effect is not observed in *Napepld*^∆IEC^ HFD mice, thereby showing a first defect in the regulation of food intake. PVN receives projections from the ARC and acts as final regulator of the hypothalamic control of anorexigenic signals^25,26^. Interestingly, although HFD feeding tends to increase c-Fos expression in the PVN (*P* = 0.06, *T*-test), *Napepld*^∆IEC^ HFD-fed mice did not respond to the diet-induced c-Fos expression in the PVN (Fig. 3e). Altogether our data show a potential defect in the integration of anorexigenic signals in the hypothalamus of HFD-fed *Napepld*^∆IEC^ mice.

Given that in basal conditions *Napepld*^∆IEC^ mice exhibited lower jejunal NAE levels, we measured the levels of different NAE in both the jejunum and in the portal blood after 4 h exposure to HFD. Interestingly, among intestinal NAE, we found that the levels of AEA are significantly decreased upon HFD exposure in WT and *Napepld*^∆IEC^ mice (Fig. 3f), whereas the concentrations of OEA are significantly increased only in WT HFD mice (*P* = 0.02, *T*-test) (Fig. 3f). It is worth noting that 2-AG, described as an orexigenic mono-acylglycerol, is significantly decreased following HFD exposure (Fig. 3g). On the contrary, other mono-acylglycerols including those exhibiting anorexigenic effects were increased during HFD exposure, and were not affected by *Napepld* deletion (Fig. 3g), thereby excluding the potential role of these mediators in the present context.

Interestingly, during HFD exposure, *Napepld*^∆IEC^ mice displayed significantly lower AEA, LEA, and PEA levels (*P* = 0.04, *T*-test) in the portal blood (Fig. 3h). The portal levels of different mono-acylglycerols were only affected by HFD exposure and not by *Napepld* deletion (Fig. 3i). Therefore, these results strongly suggest that the increased food intake is not mediated through the modulation of gut hormones but likely through intestinal bioactive lipids produced by NAPE-PLD.

### *Napepld*^∆IEC^ worsens obesity and reduces energy expenditure

**Fig. 4:**
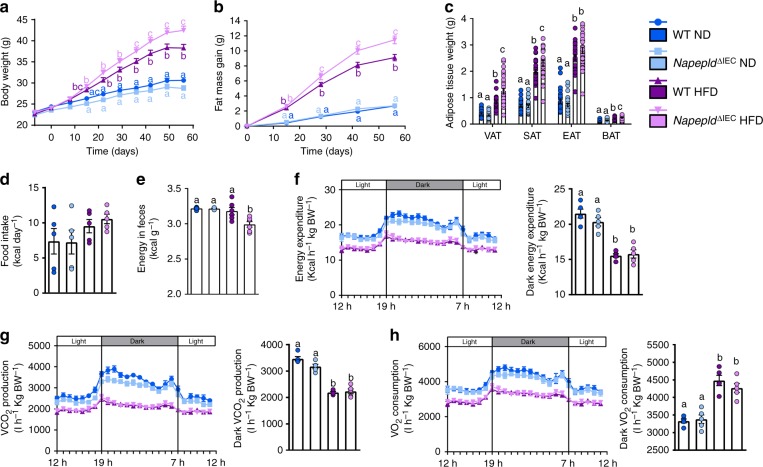
Exacerbation of HFD-induced obesity in *Napepld*^*∆IEC*^ mice. **a** Body weight (g) over an 8 weeks period. **b** Fat mass gain (g) over an 8 weeks period. **c** Weight of different white adipose tissue depots and BAT (g). **d** Daily food intake (Kcal/day) measured in metabolic chambers during indirect calorimetry studies at the 8th week of HFD feeding. **e** Energy measured in the feces at the 8th week of HFD feeding (Kcal g feces^−1^). **f** Light and dark cycle energy expenditure (Kcal h^−1^ Kg body weight^−1^) measured in metabolic chambers. **g** Light and dark cycle of CO_2_ production (l h^−1^ Kg body weight^−1^). **h** Light and dark cycle of O_2_ consumption (l h^−1^ kg body weight^−1^). Data in **a**–**c** correspond to the results of three independent experiments. For **a**–**c**, *n* = 26–28. For **d**, **f**–**h**, *n* = 5. For **e**, *n* = 4–6. Dark blue: WT ND mice, light blue: *Napepld*^∆IEC^ ND mice, purple: WT HFD mice and pink: *Napepld*^∆IEC^ HFD mice. Data are presented as the mean ± s.e.m. Data with different superscript letters are significantly different (*P* < 0.05) according to repeated measures two-way ANOVA followed by Tukey’s post-hoc test (**a**, **b**) or regular two-way ANOVA followed by Tukey’s post-hoc test (**c**, **h**)

To explore the impact of *Napepld*^∆IEC^ upon chronic fat intake, we exposed mice to HFD for 8 weeks. HFD-fed *Napepld*^∆IEC^ mice displayed significantly higher body weight (Fig. 4a) and fat mass gain (Fig. 4b) than WT mice, whereas no differences could be observed in ND conditions. Consistently, visceral (VAT), subcutaneous (SAT) and brown adipose tissue (BAT) weights were significantly higher in HFD-fed *Napepld*^∆IEC^ mice with a similar trend for the epididymal adipose depot (EAT) (Fig. 4c). As observed upon ND (Fig. 1a), *Napepld* gene expression was reduced in the jejunum and colon of *Napepld*^∆IEC^ HFD mice (Supplementary Figure 1A). The deletion was confirmed at the protein level (Supplementary Figure 1B). *Napepld*^∆IEC^ mice did not exhibit any change in intestinal expression of other key ECS actors, such as *Naaa*, *Mgl*, *Faah* or *Cnr1*. A modest increase in *Naaa* and *Mgl* expression was only observed in HFD-fed mice (Supplementary Figure 1C).

The higher fat mass gain observed in *Napepld*^∆IEC^ mice might be partially explained by both a 10% higher energy intake (*P* > 0.05, *T*-test) (Fig. 4d) and a significantly lower energy excretion in the feces (Fig. 4e), but not by a modification of whole body energy expenditure (Fig. 4f), CO_2_ production (Fig. 4g) or O_2_ consumption (Fig. 4h).

**Fig. 5:**
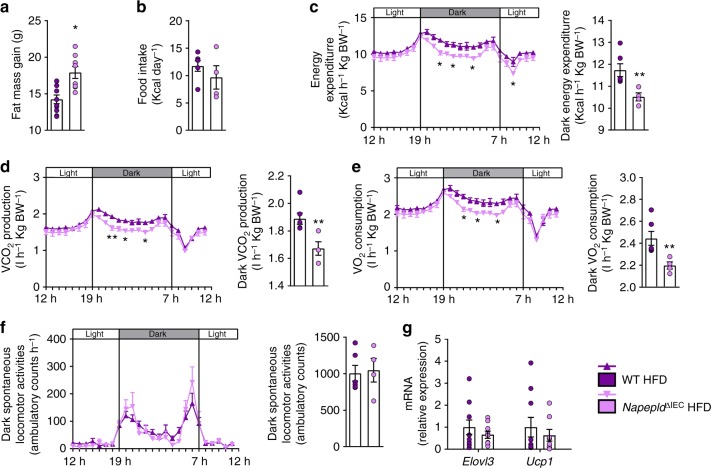
Phenotype of Napepld^*∆IEC*^ mice after 16weeks is associated with lower energy expenditure. **a** Fat mass gain (g) after a 16 weeks period. **b** Daily HFD intake (Kcal/day) measured in metabolic chambers during indirect calorimetry studies at the 16th week of HFD feeding. **c** Light and dark cycle energy expenditure (Kcal h^−1^ Kg body weight^−1^) measured in metabolic chambers. **d** Light and dark cycle of CO_2_ production (l h^−1^ Kg body weight^−1^). **e** Light and dark cycle of O_2_ consumption (l h^−1^ kg body weight^−1^). **f** Light and dark cycle spontaneous locomotor activities (Ambulatory Counts h^−1^). **g** mRNA expression of *Elovl3* and *Ucp1* in the subcutaneous adipose tissue. For **a**–**f**, *n* = 8–10. For **g**, *n* = 8–10. Purple: WT HFD mice and pink: *Napepld*^∆IEC^ HFD mice. Data are presented as the mean ± s.e.m. Asterisks (* and **) indicate a significant difference versus WT HFD (Respectively *P* < 0.05 and *P* < 0.01) according to Mann–Whitney test or repeated measures two-way ANOVA followed by Bonferroni’s post-hoc test

One could expect that the difference in fat mass would decrease after longer times of HFD. We thus assessed the phenotype of *Napepld*^*∆IEC*^ mice after a prolonged HFD exposure (16 weeks). Although no major differences in NAE and mono-acylglycerol levels could be observed in the jejunum (except for 2-PG) and in the portal blood (Supplementary Figure 3B–E), we found a significantly higher fat mass gain in *Napepld*^∆IEC^ HFD-fed mice as compared to WT HFD-fed mice (Fig. 5a). To assess the cause of this persisting fat mass difference, we investigated energy metabolism during the last week of HFD exposure. At that point, *Napepld*^∆IEC^ mice did not eat more than WT mice (Fig. 5b). However, during dark phase, *Napepld*^∆IEC^ mice spent significantly less energy than WT mice (Fig. 5c), together with lower CO_2_ production (Fig. 5d) and O_2_ consumption (Fig. 5e). It is worth noting that this was not due to any modification of the physical activity (Fig. 5f). Finally, the respiratory exchange ratio (RER) was unaffected (Supplementary Figure 3A). To further decipher the mechanisms explaining the higher energy storage, we investigated the impact of the *Napepld*^∆IEC^ deletion on adipose tissue browning processes. We have previously shown that adipocyte-specific *Napepld* deletion is associated with decreased energy expenditure and browning^11^. Here, we found a downregulation of key browning markers namely *Ucp1* and *Elovl3* in the white adipose tissue of *Napepld*^∆IEC^ mice as compared to WT HFD-fed mice, but this effect did not reach significance (Fig. 5g). Nevertheless, because this measure is obtained at the end of the experiment, we may not rule out that because of the overall higher fat mass in these mice, the trends observed here could partially contribute to the reduction of energy expenditure however this assumption warrants further investigation. Altogether, these results suggested that *Napepld*^∆IEC^ causes an increased food intake at the beginning of a HFD, leading to an increased body weight and fat mass that is maintained in the long term because of changes in energy expenditure.

### *Napepld*^∆IEC^ exacerbates diet-induced steatosis

**Fig. 6:**
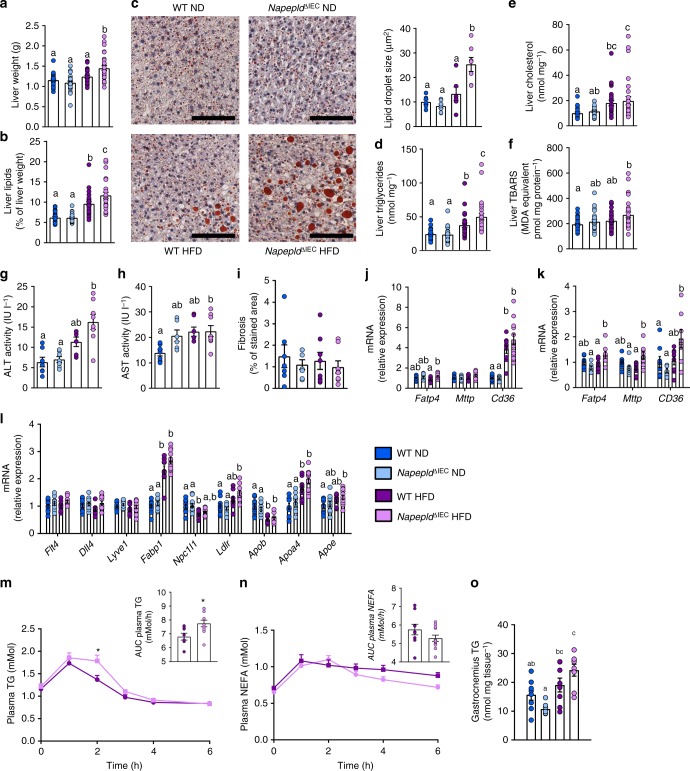
IEC-specific Napepld exacerbates diet-induced liver steatosis. **a** Liver weight (g). **b** Liver lipid content measured by gravimetry (as percentage of liver weight). **c** Representative liver Oil red O staining (scale bar: 100 µm) and mean lipid droplet size (µm², *n* = 5–7). **d** Liver triglycerides (nmol mg^−1^). **e** Liver cholesterol (nmol mg^−1^). **f** Liver thiobarbituric acid-reactive species (TBARS, pmol mg protein^−1^). **g** Plasma alanine aminotransferase (ALT) activity measured in the cava vein (IU l^−1^) (*n* = 6–8). **h** Plasma aspartate aminotransferase (AST) activity measured in the cava vein (IU l^−1^) (*n* = 6–8). **i** Histological fibrosis analysis using Sirius red staining expressed in % of area stained (*n* = 7–8). **j** mRNA expression of *Fatp4*, *Mttp*, and *Cd36* in the duodenum (*n* = 9–12). **k** mRNA expression of *Fatp4*, *Cd36*, and *Mttp* in the liver (*n* = 7–8). **l** mRNA expression of *Flt4*, *Dll4*, *Lyve1, Fabp1*, *Npc1l1*, *Ldlr*, *Apob*, *Apoa4* and *Apoe* in the duodenum (*n* = 9–12). **m** Plasma TG in the caudal vein after a lipid load challenge (300 μl of Intralipid 20% emulsion) (mMol), Inset: area under the curve (AUC) of TG level evolution during lipid load test (*n* = 7–12). **n** Plasma NEFA in the caudal vein after a lipid load challenge (300 μl of Intralipid 20% emulsion) (mMol), Inset: area under the curve (AUC) of NEFA level evolution during lipid load test (*n* = 7–12). **o** Gastrocnemius TG content (nmol mg of tissue^−1^) (*n* = 7–8). *n* = 26–28 for **a**, **b**, and **d**–**f**. Data **a**, **b**, and **d**–**f** correspond to the results of three independent experiments. Dark blue: WT ND mice, light blue: *Napepld*^∆IEC^ ND mice, purple: WT HFD mice and pink: *Napepld*^∆IEC^ HFD mice. Data are presented as the mean ± s.e.m. Data with different superscript letters are significantly different (*P* < 0.05) according to regular two-way ANOVA followed by Tukey’s post-hoc test. Asterisk (*) indicates a significant difference versus WT HFD (*P* < 0.05) according repeated measures two-way ANOVA followed by Bonferroni’s post-hoc test

Aside from the fat depots, liver weight was also significantly higher in HFD-fed *Napepld*^∆IEC^ mice than in all other groups after 8 weeks of HFD feeding (Fig. 6a). This was associated with a worsening of HFD-induced steatosis, as total hepatic lipid content, hepatic triglycerides and lipid droplet size were significantly higher in *Napepld*^∆IEC^ mice than in WT mice (Fig. 6b–d). This was supported by a trend for higher gene expression of lipid synthesis genes including *Fasn*, *Acaca*, *Scd1, Srebf1, Me1, Gpam,* and *Dgat2* (Supplementary Figure 4A), and no changes in genes involved in lipid oxidation such as *Ppargc1a, Ppara, Cpt1a, Acox, and Acadm* (Supplementary Figure 4B). In accordance with the gene expression profile, levels of Thr^172^ p-AMPK were similar between groups (Supplementary Figure 4H). Hepatic cholesterol was only affected by HFD (Fig. 6e), as were plasma cholesterol levels (Supplementary Figure 4C). Plasma triglycerides and non-esterified fatty acids (NEFA) were unchanged in all groups (Supplementary Figure 4D and E). Lipid peroxidation was significantly increased in HFD-fed *Napepld*^∆IEC^ mice when compared to ND-fed animals (Fig. 6f). As compared to WT ND, plasma alanine aminotransferase tended to be increased in WT HFD mice and was significantly increased in HFD-fed *Napepld*^∆IEC^ (Fig. 6g). Plasma aspartate aminotransferase was doubled in both HFD-fed *Napepld*^∆IEC^ mice (*P* < 0.05, *T*-test) and in WT HFD (*P* > 0.05, *T*-test) (Fig. 6h). Finally, HFD-fed *Napepld*^∆IEC^ mice also displayed trends for increased expression of genes encoding collagen fibers associated with steatohepatitis development^27^ (Supplementary Figure 4F), but this effect was not associated with higher fibrosis (Fig. 6i). These data suggest that *Napepld*^∆IEC^ mice are more sensitive to diet-induced obesity, liver steatosis, and oxidative stress. We next investigated the mechanisms involved in the development of steatosis, by focusing our attention on several factors. Hepatic steatosis may occur through increased AEA levels^20,28^ together with hepatic CB1 over-activity during HFD feeding^28^. However, liver *Cnr1* mRNA expression was unaffected in all conditions (Supplementary Figure 4G). Given that the steatotic phenotype is not explained by a specific modification of lipid synthesis or oxidation, we investigated several markers of both intestinal and hepatic lipid absorption. We found a significantly higher expression of *Fatp4* in the proximal gut of *Napepld*^∆IEC^ mice exposed to HFD compared to WT HFD-fed mice (Fig. 6j). A similar trend was observed for *Cd36* and *Mttp* that were increased by about 25% in *Napepld*^∆IEC^ mice upon HFD (Fig. 6j). Consistently, *Fatp4* and *Cd36* were significantly increased in the liver of *Napepld*^∆IEC^ mice (Fig. 6k). To extend this finding, we analyzed additional markers of lipid metabolism in the duodenum. As intestinal lipid absorption mainly occurs through the lacteals^29^, we measured lacteals markers (*Flt4* encoding Vegfr3*, Dll4*, and *Lyve1*), but they were not affected in any condition (Fig. 6l). In addition to *Fatp4* and *Cd36*, we also measured other markers of fat absorption and lipoprotein metabolism in the duodenum. Among these, *Fabp1, Ldlr, Apoa4* and *Apoe* mRNA expression were significantly increased in *Napepld*^∆IEC^ HFD fed mice as compared to WT ND, whereas a trend was observed compared to WT HFD (Fig. 6l).

To demonstrate the putative implication of *Napepld*^∆IEC^ in lipid absorption, we investigated the impact of an acute oral lipid load. As expected, oral lipid administration increased plasma triglycerides levels in both WT HFD and *Napepld*^∆IEC^ HFD fed mice. However, *Napepld*^∆IEC^ mice exhibited significantly higher triglycerides levels as depicted by both the lipid profile (higher levels after 2 h) and the area under the curve (Fig. 6m). In addition, the levels of NEFA tend to decrease faster in the *Napepld*^∆IEC^ HFD mice than in WT HFD mice (Fig. 6n), suggesting higher storage of fat. Besides the higher liver and adipose tissue fat storage, we also noticed an ectopic deposition of triglycerides in the muscles of *Napepld*^∆IEC^ HFD-fed mice, with a significantly 2-fold higher lipid content as compared to WT ND mice and a trend of 27% higher fat content than in WT HFD mice (Fig. 6o).

Altogether, our data strongly suggest that the increased lipid storage observed in the liver and in other tissues (i.e., adipose tissue and muscles) is likely explained by an increased capacity to absorb fatty acids in these organs. These results are also consistent with the lower energy excretion in the feces of *Napepld*^∆IEC^ HFD fed mice (Fig. 4e).

### *Napepld*^∆IEC^ does not affect HFD-induced adipose inflammation

Increased VAT mass is correlated to pro-inflammatory signals linking obesity to metabolic insults such as insulin resistance^30^. As expected, HFD feeding significantly increased the expression of all inflammatory markers investigated such as *Cd11c* (M1 macrophage marker), *Mcp1* (encoding the Monocyte chemoattractant protein 1), *Serpin1* (encoding Plasminogen Activator Inhibitor-1, PAI-1) and *Lbp* (encoding Lipopolysaccharide Binding Protein, LBP) (Supplementary Figure 5). All these markers were further increased by about 25% in HFD-fed *Napepld*^∆IEC^ mice, but this effect did not reach significance (Supplementary Figure 5). *Mcp1* was significantly higher in ND-fed *Napepld*^∆IEC^ mice suggesting an increased pro-inflammatory tone in *Napepld*^∆IEC^ mice even in the absence of a HFD.

### *Napepld*^∆IEC^ does not affect glucose metabolism

**Fig. 7:**
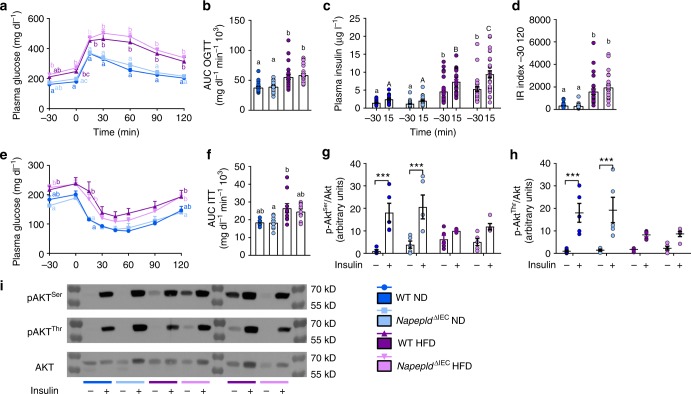
IEC-specific Napepld deletion does not affect glucose homeostasis. **a** Plasma glucose profile (mg dl^−1^) measured between 30 min before and 120 min after glucose loading (*n* = 26–28). **b** Mean area under the curve (AUC, mg dl^−1^ min^−1^ 10^3^) measured between 30 min before and 120 min after glucose loading (*n* = 26–28). **c** Plasma insulin levels (µg l^−1^) at 30 min before and 15 min after glucose loading (*n* = 26–28). **d** Insulin resistance index determined by multiplying the AUC of blood glucose by the AUC of insulin (*n* = 26–28). **e** Plasma glucose profile (mg dl^−1^) measured between 30 min before and 120 min after insulin injection. **f** Mean area under the curve (AUC, mg dl^−1^ min^−1^ 10^3^) measured between 30 min before and 120 min after insulin injection (*n* = 5–11). **g**, **h** Ratio of the vehicle- and insulin-stimulated **g** p-Akt ^ser473^ and **h** p-Akt^thr308^ on total Akt measured by densitometry. **i** Representative western-blot for hepatic p-Akt^thr308^, p-Akt^ser473^, and Akt with or without insulin stimulation (*n* = 9–11). Dark blue: WT ND mice, light blue: *Napepld*^∆IEC^ ND mice, purple: WT HFD mice and pink: *Napepld*^∆IEC^ HFD mice. Data in **a**–**d** correspond to the results of three independent experiments. Data are presented as the mean ± s.e.m. Data with different superscript letters are significantly different (*P* < 0.05) according to repeated measures two-way ANOVA followed Tukey’s post-hoc test (**a**, **c**, **e**) or regular two-way ANOVA followed by Tukey’s post-hoc test (**b**, **d**, **f**). Asterisk (***) indicates a significant difference versus vehicle-injected group (*P* < 0.001) according to two-way ANOVA followed by Sidak’s post-hoc test (**g**, **h**)

Glucose metabolism was mostly affected by the HFD, leading to significant hyperglycemia and hyperinsulinemia at all time points during an oral glucose tolerance test (Fig. 7a–c). A significantly increased insulinemia could be observed 15 min after the glucose challenge in HFD-fed *Napepld*^∆IEC^ mice (Fig. 7c). The insulin resistance index was mostly increased by HFD feeding and tended to be higher in HFD-fed *Napepld*^∆IEC^ mice compared to WT HFD mice (Fig. 7d). The increased glucose-induced hyperinsulinemia observed in *Napepld*^∆IEC^ mice prompted us to verify insulin sensitivity with an insulin tolerance test. As anticipated, HFD led to a significant insulin resistance, but this was not exacerbated in *Napepld*^∆IEC^ mice (Fig. 7e, f). Finally, we investigated the activation of the insulin signaling pathway in the liver after portal vein insulin administration. HFD strongly blunted the phosphorylation of hepatic p-Akt^thr308^ and p-Akt^ser473^, revealing the presence of massive hepatic insulin resistance. This defect was however similar in *Napepld*^∆IEC^ mice (Fig. 7g–i).

All the aforementioned results confirm that the exacerbation of the obese phenotype of *Napepld*^∆IEC^ mice under HFD condition is not likely linked with an alteration of glucose metabolism. However, the higher insulinemia observed post oral glucose challenge is a phenomenon that can contribute in itself at least partially to higher lipid storage^31,32^.

### *Napepld*^∆IEC^ alters the gut microbiota

**Fig. 8:**
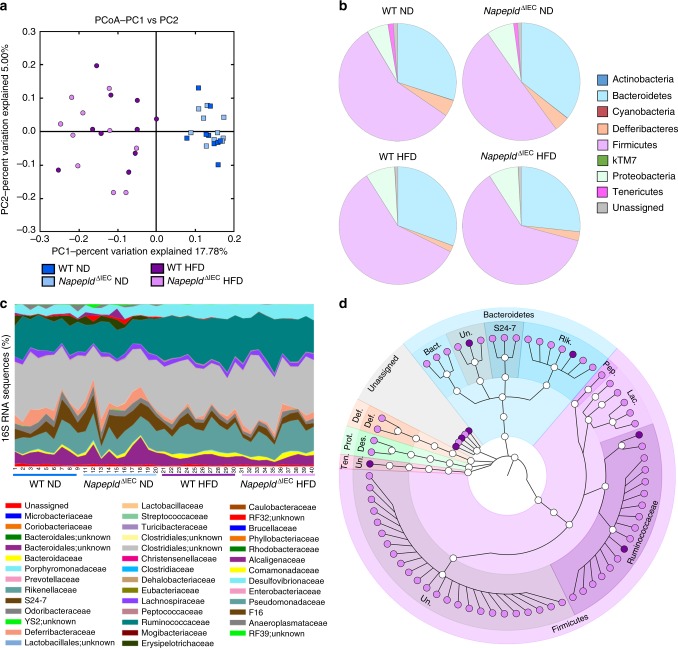
Effects of IEC *Napepld* deletion on the gut microbiota. **a** Principal coordinates analysis (PcoA) based on the unweighted UniFrac analysis on operational taxonomic units (OTUs). Each symbol representing a single sample is colored according to its group. **b** Composition of abundant bacterial phyla identified in the gut microbiota. **c** Relative abundances (percentage of 16S rRNA gene sequences) of the different bacterial families in each sample. **d** OTUs significantly affected in HFD-fed *Napepld*^∆IEC^ mice versus WT mice. A representative 16S rRNA gene from each of the 75 differentially expressed OTUs was aligned and used to infer the phylogenetic tree shown in this figure (*n* *=* 9–10). Purple: WT HFD mice and pink: *Napepld*^∆IEC^ HFD mice. Ten., Tenericutes; Prot., Proteobacteria; Def., Deferribacteres; Bact., Bacteroidaceae; Rik., Rikenellaceae; Pep., Peptococcaceae; Lac., Lachnospiraceae; Des., Desulfovibrionaceae; Def., Deferribacteraceae; Un., Unknown

The intestinal ECS can contribute to energy homeostasis regulation through modulation of the gut microbiota^3,33^. We previously found that the gut microbiota interacts with the ECS and changes the expression of *Cnr1*, *Cnr2* (encoding CB2) and enzymes including *Napepld*^3^ in the intestine. Conversely, disruption of the ECS mediated by adipocyte-specific *Napepld* deletion alters gut microbiota composition and causes obesity in ND-fed mice^11^. We therefore analyzed the microbiota of our mice by metagenomics analysis. As expected, principal coordinates analysis (PCoA) showed that HFD drastically changed gut microbiota composition as shown by the shift on the first axis (Fig. 8a). Consistent with previous studies, HFD significantly affected the proportion of different phyla^34,35^. HFD-fed mice presented an increase in Proteobacteria and a decrease in Deferribacteres, Tenericutes, and kTM7 (Fig. 8b and Supplementary Table 1). Fifteen bacterial families were significantly affected by HFD (Fig. 8c and Supplementary Table 2). At the genus level, 19 bacterial genera were significantly modified by HFD (Supplementary Data 1) with 11 bacterial genera remaining significantly affected after correction by a false discovery rate (FDR) test according to the Benjamini–Hochberg procedure. *Napepld*^∆IEC^ slightly affected gut microbiota composition in ND-fed mice (Fig. 8c and Supplementary Tables 1, 2 and Supplementary Data 1). We did not find major differences between HFD-fed WT and *Napepld*^∆IEC^ mice at the phylum, class or genus level (Fig. 8c and Supplementary Tables 1, 2 and Supplementary Data 1). However, the relative abundance of 75 operational taxonomic units (OTUs) was significantly different between HFD-fed *Napepld*^∆IEC^ mice and WT mice, thereby suggesting a strong and specific alteration of the microbiota composition due to the *Napepld* deletion and not due to the HFD feeding per se (Fig. 8d).

### *Napepld*^∆IEC^ does not alter benefits of *A. muciniphila*

**Fig. 9:**
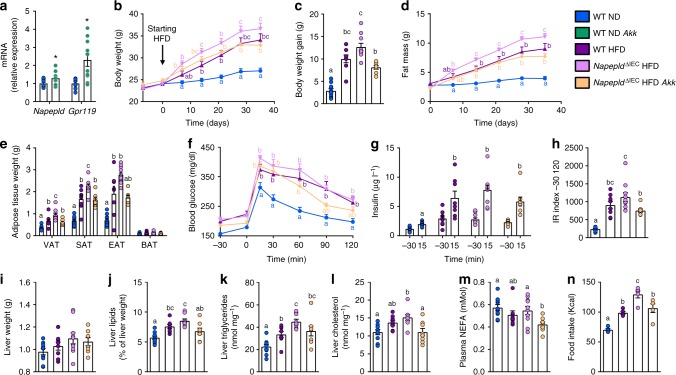
Effects of *A. muciniphila* in HFD-fed *Napepld*^∆IEC^ mice. **a** mRNA expression of *Napepld* and *Gpr119* in the ileum of ND-fed mice upon treatment with *A. muciniphila*. **b** Body weight (g) over a 5 weeks period. **c** Body weight gain (g) after 5 weeks. **d** Fat mass (g) over a 5 weeks period. **e** Weight of different white adipose tissue depots and BAT (g). **f** Plasma glucose profile (mg dl^−1^) measured between 30 min before and 120 min after glucose loading. **g** Plasma insulin levels (µg l^−1^) at 30 min before and 15 min after glucose loading. **h** Insulin resistance index determined by multiplying the AUC of blood glucose by the AUC of insulin. **i** Liver weight (g). **j** Liver lipid content (as percentage of liver weight) measured by gravimetry. **k**) Liver triglycerides (nmol mg^−1^). **l** Liver cholesterol (nmol mg^−1^). **m** Plasma non-esterified fatty acids (NEFA, mMol). For **a**–**m**, *n* = 8–10. **n** Food intake during the first week (kcal, *n* = 5). See also Supplementary Figure 4. Dark blue: WT ND mice, green: WT ND *Akk* mice, purple: WT HFD mice, pink: *Napepld*^∆IEC^ HFD mice and orange: *Napepld*^∆IEC^ HFD *Akk* mice. Data are presented as the mean + s.e.m. Asterisk (*) indicates a significant difference versus WT ND (*P* < 0.05) according to Student’s *t*-test. Data with different superscript letters are significantly different (*P* < 0.05) according to one-way ANOVA followed by Tukey’s post-hoc test (**c**, **e**, **h**–**n**) or repeated measures two-way ANOVA followed by Tukey’s post-hoc test (**b**, **d**, **f**, **g**)

Metabolic alterations of HFD-fed *Napepld*^∆IEC^ mice are associated with changes in gut microbiota composition. We wondered whether targeting the gut microbiota of *Napepld*^∆IEC^ mice could correct their phenotype. We and others previously showed that *A. muciniphila* protects mice against diet-induced obesity and can reduce body weight gain even in control diet-fed mice^36,37^. Furthermore, *A. muciniphila* can modulate intestinal levels of eCB and related mediators in HFD-fed mice^36^. Interestingly, *A. muciniphila* treatment increased ileal expression of *Napepld* and *Gpr119*, encoding one of the receptor activated by OEA-stimulated gut peptides controlling appetite^5^ (Fig. 9a). This suggests that NAPE-PLD and its products might be involved in the dialog between *A. muciniphila* and its host. We tested this hypothesis by administering the bacterium to HFD-fed *Napepld*^∆IEC^ mice during 5 weeks. This time point was chosen as it is sufficient to observe significant differences between WT and *Napepld*^∆IEC^ mice (Fig. 4a, b) as well as significant impacts of *A. muciniphila* in HFD-fed mice^34^. As expected, *Napepld*^∆IEC^ mice gained more body weight and fat mass than WT animals after 5 weeks of HFD feeding (Fig. 9b–d). White adipose tissue weights were also higher in *Napepld*^∆IEC^ mice, although BAT weight was similar in all conditions (Fig. 9e).

*A. muciniphila* treatment significantly reduced body weight and fat mass in *Napepld*^∆IEC^ mice (Fig. 9b–e). It also tended to decrease hyperglycemia and hyperinsulinemia during an OGTT, leading to a significantly lower insulin resistance index (Fig. 9f–h and Supplementary Figure 6A). Five weeks of treatment were not sufficient to induce changes in liver weight (Fig. 9i) though HFD-induced steatosis was already present and tended to be higher in *Napepld*^∆IEC^ mice (Fig. 9j). This was associated with significantly higher triglyceride content and a similar trend for cholesterol (Fig. 9k, l). *A. muciniphila* treatment reduced steatosis in HFD-fed *Napepld*^∆IEC^ mice with significant decreases in liver lipid and cholesterol content and a similar trend for triglycerides (Fig. 9j–l). *A. muciniphila* administration also significantly lowered NEFA levels in HFD-fed *Napepld*^∆IEC^ mice when compared to all other groups (Fig. 9m). *A. muciniphila* corrected the hyperphagia observed in *Napepld*^∆IEC^ mice during the first week of HFD exposure (Fig. 9n). Finally, *A. muciniphila* did not affect ileal expression of *Napepld*, *Gpr119*, *Cnr1*, and *Ppara* in *Napepld*^∆IEC^ HFD-fed mice (Supplementary Figure 6B). Altogether, these data suggest that intestinal NAPE-PLD, and possibly its NAE products, are not needed for the protective effects of *A. muciniphila* against obesity and the metabolic syndrome.

**Fig. 10:**
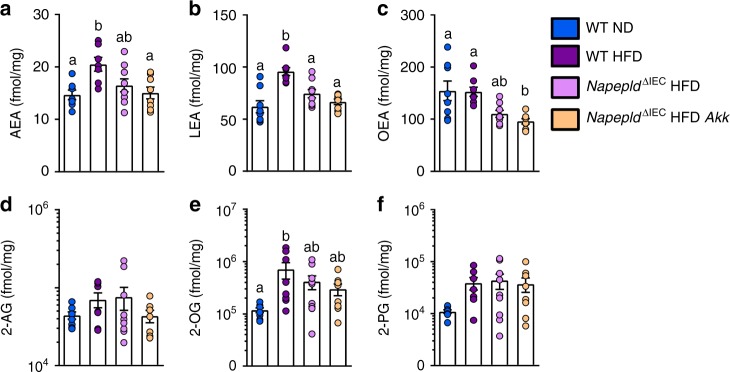
Effects of *Napepld*^∆IEC^ and *A. muciniphila* on the levels of bioactive lipids in the jejunum. **a** AEA, **b** LEA, **c** OEA, **d** 2-AG, **e** 2-OG, **f** 2-PG (fmol/mg) measured in the jejunum using Shimadzu 8050 triple quadrupole mass spectrometer. *n* = 7–9. Dark blue: WT ND mice, purple: WT HFD mice, pink: *Napepld*^∆IEC^ HFD mice and orange: *Napepld*^∆IEC^ HFD *Akk* mice. Data are presented as the mean + s.e.m. Data with different superscript letters are significantly different (*P* < 0.05) according to one-way ANOVA followed by Tukey’s post-hoc test

We previously observed that *A. muciniphila* administration affects the levels of 2-AG, and of its monoacylglycerol congeners, 2-OG and 2-PG in the distal small intestine of HFD-fed mice^36^; however, its impact on the proximal small intestine was unknown. Here we found that 5 weeks of HFD, as opposed to what is shown above for a 4 h HFD, significantly increased AEA and LEA, and tends to increase 2-AG in the jejunum. Similar to what was observed for a 4 h HFD, 2-OG, and 2-PG levels were also increased (Fig. 10). Conversely, and unsurprisingly, *Napepld*^∆IEC^ mice on a HFD exhibited a decrease in some NAE levels (i.e., a significant decrease for LEA and a similar trend for OEA and AEA), and this effect was not significantly affected by treatment with *A. muciniphila* (Fig. 10). This finding supports the role of intestinal NAPE-PLD in the production of these mediators in the upper part of the gut during HFD. Interestingly, treatment with *A. muciniphila* of *Napepld*^∆IEC^ *mice* did not significantly affect the levels of 2-AG and other 2-acylglycerols, unlike what previously observed in the lower tract of WT HFD mice (33).

## Discussion

Our results show that intestinal *Napepld* acts as a master sensor for dietary fat in the gut-to-brain axis contributing to the onset of HFD-induced metabolic disorders. Previous reports have shown that specific NAE such as OEA or AEA are affected by dietary lipids in the gastrointestinal tract^17,21^, and linked variations in their levels with modulation of appetite and satiety. However, the origin of such variation as well as the impact of a long-term HFD exposure were unknown.

Here, we show that intestinal epithelial *Napepld* plays an important role in the regulation of food intake upon the first HFD exposure and eventually the long-term regulation of energy homeostasis. Moreover, we discovered that besides its impact on food intake and fat mass gain, intestinal *Napepld* is a key enzyme protecting against the onset of HFD-induced hepatic steatosis. Our data suggest that intestinal NAE produced via NAPE-PLD modulate energy absorption, storage and expenditure, and that disruption of this signaling system leads to the development of obesity and hepatic steatosis.

Intestinal epithelial *Napepld* deletion does not affect mice fed a standard chow diet. Conversely, as soon as *Napepld*^∆IEC^ mice were exposed to an energy-dense food such as HFD, they displayed a reduced capacity to regulate food intake and long-term energy homeostasis. This observation strongly suggests that intestinal NAPE-PLD is at least as important as specific gut hormones for controlling food intake.

Levels of the eCB, AEA, and other non-eCB NAE, partly depend on the activity of NAPE-PLD^38^. Accordingly, we found a decrease in AEA, OEA, PEA, SEA, LEA, with no changes in the mono-acylgycerols biochemically related to the other eCB, with the exception of 2-AG, which was also decreased in the jejunum of Napepld^∆IEC^ mice. This finding was surprising since this compound is not biosynthesized by NAPE-PLD. This decrease might be explained by the decrease in PEA, which was shown to enhance 2-AG levels under various experimental conditions^39,40^. In order to investigate if other bioactive lipids or precursors were affected in *Napepld*^∆IEC^ mice, we performed a broad lipidomic analysis (Supplementary Tables 5–10) in the intestine by measuring lysophosphatidylethanolamines, phosphatidylethanolamines, lysophosphatidylcholine, lysophosphatidylglycerols, phosphatidylglycerols, phosphatidylserines, phosphatidylcholines, ceramides, and sphyngomyelins. Strikingly, besides a specific impact of the HFD, none of these lipids were affected by the *Napepld* deletion, thereby suggesting that the phenotype of *Napepld*^∆IEC^ mice is caused by changes in NAE levels. Moreover, no potential compensatory mechanisms involving *Naaa, Faah, Mgl*, or *Cnr1* were observed in the gut of *Napepld*^∆IEC^ mice.

HFD exposure is known to induce several factors modulating food intake and energy homeostasis such as gut hormones (e.g., ghrelin and GLP-1) and hypothalamic neurotransmitters (e.g., *Pomc, Cart, Npy, Agrp*)^41^. In this study, we excluded the role of gut peptides since ghrelin and GLP-1 were only affected by HFD feeding. However, in *Napepld*^∆IEC^ mice, *Pomc* expression was not induced by HFD exposure. Moreover, we found that HFD feeding tends to not increase POMC activation in HFD-fed *Napepld*^∆IEC^ mice. Interestingly, we also discovered that *Napepld*^∆IEC^ mice did not respond to the HFD-induced neuronal activation in the PVN. These data show that *Napepld*^∆IEC^ mice display an alteration in the gut-to-ARC-to-PVN neuronal signaling. Finally, HFD exposure also affected NAE levels in both the jejunum and in the portal blood of in *Napepld*^∆IEC^ mice, thereby suggesting the involvement of intestinal NAE in the control of dietary fat intake and the regulation of hypothalamic neurons. This finding also shows that intestinal NAE could modulate the activity of hypothalamic anorectic neurons within a short period of time. This association between defective *Pomc* regulation and exacerbated diet-induced obesity in *Napepld*^∆IEC^ mice is in line with results obtained in outbred mice, in which dysregulation of *Pomc* expression was the earliest factor differentiating obesity-prone and obesity-resistant animals upon HFD feeding^23^. Hyperphagia was only observed in HFD-fed *Napepld*^∆IEC^ mice during the first week of feeding. Despite the absence of change in energy expenditure, *Napepld*^∆IEC^ mice already displayed lower fecal energy excretion and higher fat absorption. Altogether, these data suggest that the increased fat mass, muscles lipid content and hepatic steatosis might be at least partially explained by increased fatty acid retention. Conversely, despite an absence of significant alterations in both intestinal and portal blood NAE, the sustained increase in fat mass and eventually body weight was likely due to a second mechanism, which is the lower energy expenditure, which was still observed after 16 weeks of HFD exposure, and a trend for impaired browning processes. These data are consistent with the effects observed in mice lacking *Napepld* in the adipose tissue that are characterized by a lower energy expenditure and browning of the adipose tissue^11^.

In accordance with our observation reinforcing the key role played by intestinal *Napepld*, previous data have shown that fat intake and its passage through the stomach and proximal small intestine lead to the synthesis of NAE to fine-tune food intake between meals^17,18^. As NAE synthesis is decreased in *Napepld*^∆IEC^ mice, this fine-tuning is disrupted, which could account for their hyperphagia. Upon prolonged HFD feeding, an exacerbation of HFD-induced reduction in energy expenditure is observed in *Napepld*^∆IEC^ mice and could explain the maintenance of the phenotype over time.

*Napepld* deletion in both ND- and HFD-fed mice was found to reduce the levels of all NAE. However, it is tempting to speculate that the phenotype observed in HFD-fed *Napepld*^∆IEC^ mice is due to changes in PEA, LEA, SEA and/or OEA, rather than AEA levels, since the intestinal abundance of most of these NAE is higher than than AEA. Further, LEA and OEA can activate targets that inhibit food intake and fat accumulation, while AEA stimulates opposite effects. This would be in agreement with the fact that reduced, rather than elevated, non-eCB NAE levels in the small intestine are associated with hyperphagia and obesity in rodents^42,43^.

In addition to body weight and fat mass gain, *Napepld*^∆IEC^ exacerbates hepatic steatosis in HFD-fed mice. The transient hyperphagia in *Napepld*^∆IEC^ mice is probably not sufficient to explain this higher lipid storage, and other factors affected in *Napepld*^∆IEC^ mice are likely to be involved. More specifically, *Napepld*^∆IEC^ mice absorb more lipids in the gut and liver while excreting less energy in the feces. Mice also develop hyperinsulinemia, which can also contribute to hepatic lipid storage^31,32^. Consistently, the expression of lipogenic enzymes (*Fasn, Acaca, Scd1, Srebf1, Me1*) only tended to be higher in HFD-fed *Napepld*^∆IEC^ mice, without changes in the phosphorylation status of AMPK whereas *Napepld*^∆IEC^ mice exhibited an increased expression of hepatic and intestinal lipid transporters. These results were further confirmed upon an oral lipid load thereby suggesting that intestinal *Napepld* regulates lipid absorption. Absence of intestinal *Napepld* thus leads not only to hepatic steatosis but also to other ectopic fat deposition (e.g., muscles). Although increased AEA and CB1 expression levels have been described during steatosis^20,28^, suggesting a role for hepatic CB1 over-activity in the process^28^, we did not find any changes in *Cnr1* expression in our model, thereby excluding such potential mechanism of action.

The gut microbiota directly regulates the intestinal ECS^11,44^. Although all mice were littermate and *Napepld* deletion was induced in adult mice, it still led to specific changes in the gut microbiota of *Napepld*^∆IEC^ mice. Several OTUs significantly differed between HFD-fed WT and *Napepld*^∆IEC^ mice, indicating a potential crosstalk between intestinal NAE and gut microbes. Whether these changes contribute to the phenotype of HFD-fed *Napepld*^∆IEC^ mice is not clear at this point and should be further investigated. However, the modulation of the gut microbiota and the use of specific bacteria as beneficial microbes represent an exciting strategy for the management of the metabolic syndrome^45,46^. *A. muciniphila* is known to protect HFD-fed mice against the development of obesity and type 2 diabetes and stimulates the intestinal production of 2-acylglycerols^36^. Although gene expression data supported the involvement of intestinal NAPE-PLD and NAE in the interplay between *A. muciniphila* and its host, we show here that the bacterium still partly corrects HFD-induced hyperphagia and obesity in *Napepld*^∆IEC^ mice. This indicates that effects of the bacterium do not rely on IEC NAPE-PLD. We previously suggested that *A. muciniphila* exerts its beneficial effects on metabolism in part by elevating lower intestinal 2-AG and 2-acylglycerol levels (2OG and 2PG)^36^, and that the similar beneficial effects of probiotics might be due to changes in NAE intestinal levels^3^. Therefore, we measured NAE and acylglyerols in the jejunum of mice treated with a HFD and/or *A. muciniphila* (Fig. 10). Our data confirm that NAPE-PLD is required for the synthesis of several NAE but not of 2-acylglycerols in the proximal intestine. However, 2-acylglycerols were not affected by administration of the bacterium. This latter finding suggests that the effect of *A. muciniphila* on 2-acylglycerols is either restricted to the distal small intestine^36^ or requires the NAPE-PLD to occur. In any case, the fact that NAPE-PLD does not appear to be necessary for *A. muciniphila* to elicit its beneficial metabolic actions is also confirmed by our finding that administration of the bacterium blunts hyperphagia and HFD-induced hepatic steatosis as well as circulating NEFA in *Napepld*^∆IEC^ mice, uncovering new mechanisms of action of the bacterium on the metabolism of its host. It might be of interest to measure eCB and eCB-like mediators in the liver to verify whether the effects of the bacterium or the presence of NAPE-PLD changes the profile of liver bioactive lipid compounds.

In conclusion, our study highlights the intestinal NAPE-PLD as a master regulator of energy homeostasis and a metabolic sensor of dietary fat. In addition, our data show the importance of intestinal NAE in the metabolic adaptations to a lipid-rich diet. In the absence of NAPE-PLD, the severity of diet-induced obesity is more pronounced with increased accumulation of fat in the adipose tissue and liver. This is associated with alterations of hypothalamic mechanisms regulating food intake in response to dietary lipids and changes in the gut microbiota. We also show that the bacterium *A. muciniphila* is effective against diet-induced obesity even in the absence of IEC NAPE-PLD, suggesting that *A. muciniphila* could remain an interesting therapeutic tool in obese patients with alterations in NAPE-PLD expression^8^. Finally, our findings unveil the intestinal NAPE-PLD as a key molecular target to regulate intestinal fat absorption, liver lipid deposition and eventually the hypothalamic control of energy homeostasis.

## Methods

### Mice

All mouse experiments were approved by the ethical committee for animal care of the Health Sector of the Universite catholique de Louvain, under the supervision of Prof. F Lemaigre et JP Dehoux under the specific number 2010/UCL/MD/022, 2014/UCL/MD/010, 2017/UCL/MD/005 performed in accordance with the guidelines of the local ethics committee and in accordance with the Belgian Law of May 29, 2013, regarding the protection of laboratory animals (agreement number LA1230314). Mouse data are expressed as the mean ± s.e.m. Number of mice allocated per group was based on previous experiments investigating the effects of *Napepld* deletion in the adipose tissue on diet-induced obesity^11^. At the beginning of each experiment, cages were randomly assigned to experimental groups to ensure that each group was matched in terms of body weight and fat mass. No blinding procedure was followed.

Exclusion criteria were predefined as follows: mice displaying abnormal behavior (for example, increased aggressiveness leading to alteration of food intake and/or body weight loss) during the follow-up period were excluded from analyses. All tissues were carefully examined during necropsy and sampling. Any mouse displaying lesions (for example, granulous liver) was also excluded. Finally, for all analyses and for each group, any exclusion decision was supported by the use of the Grubbs test for outlier detection.

### Generation of *Napepld*^∆IEC^ mice

Inducible intestinal epithelial *Napepld*-deleted (*Napepld*^∆IEC^) C57Bl6/J mice were generated by crossing mice bearing a tamoxifen-dependent Cre recombinase expressed under the control of the villin promoter (Villin Cre-ERT2) with mice harboring a *loxP*-flanked *Napepld* allele^6,22^. All the mice used in this study are littermates and bred in specific pathogen free (SPF) animal facility. The deletion was induced at 8 weeks of age by intra peritoneal (i.p.) injection of 100 µl tamoxifen (10 mg ml^−1^) for 5 consecutive days. The control mice (WT) were injected at 8 weeks of age by intra peritoneal (i.p.) injection of 100 µl of vehicle (filtered sunflower oil with ethanol) for 5 consecutive days. Tamoxifen was prepared by addition of ethanol to 100 mg of tamoxifen (tamoxifen-free base, MP Biomedicals) to obtain a 100 mg ml^−1^ of tamoxifen suspension. A 10 mg ml^−1^ tamoxifen solution was prepared by addition of filtered sunflower oil, followed by 30 min sonication. The 10 mg ml^−1^ solution of tamoxifen solution was stored at 4 °C for up to 1 week. The tamoxifen solution was sonicated 5 min before use.

### Functional validation of *Napepld* deletion

Nine-week-old WT or *Napepld*^∆IEC^ male mice (17 mice, *n* = 9 WT, 8 *Napepld*^∆IEC^) were housed in pairs in specific pathogen free conditions and in a controlled environment (room temperature of 23 ± 2 °C, 12 h daylight cycle) with free access to sterile food (irradiated) and sterile water. The mice were fed a ND (AIN93Mi; Research diet) for 2 weeks.

### Phenotyping in ND and HFD conditions

Three independent cohorts of 9 weeks-old WT or *Napepld*^∆IEC^ male mice (first set: 40 mice, *n* = 10 per group; second set: 32 mice, *n* = 8 per group; third set: 50 mice, *n* = 12 per group except for *Napepld*^∆IEC^ HFD with *n* = 14) were housed in pairs in specific pathogen free conditions and in a controlled environment (room temperature of 23 ± 2 °C, 12 h daylight cycle) with free access to sterile food (irradiated) and sterile water. The mice were fed a ND (AIN93Mi; Research diet) or a HFD (60% fat and 20% carbohydrates (kcal per 100 g), D12492i, Research diet). Body weight, food and water intake were recorded weekly. Body composition (lean and fat mass) was assessed weekly by using 7.5 MHz time domain-nuclear magnetic resonance (TD-NMR; LF50 minispec, Bruker). Treatment continued for 8 weeks. For each cohort, an oral glucose tolerance test was performed after 7 weeks of treatment.

An additional cohort of 9 weeks-old WT or *Napepld*^∆IEC^ male mice (42 mice, *n* = 10 per ND-fed group and *n* = 11 per HFD-fed group) were housed in pairs or in cages of three in specific pathogen free conditions and in a controlled environment (room temperature of 23 ± 2 °C, 12 h daylight cycle) with free access to sterile food (irradiated) and sterile water. This cohort was followed similarly to the ones described above for 8 weeks. An insulin tolerance test was performed after 7 weeks of treatment.

### Individual measures of food intake during early HFD exposure

A cohort of 9-weeks-old WT or *Napepld*^∆IEC^ male mice (40 mice, *n* = 10 per group) was followed for 8 weeks as described above, with the following modifications: following induction of the deletion, 20 mice (10 WT and 10 *Napepld*^∆IEC^) were housed individually with free access to ND sterile food (irradiated) and sterile water. At 10-week old, mice were placed in metabolic chambers (Labmaster, TSE Systems GmbH, Bad Homburg, Germany) for individual measurement of food intake. For the next 8 weeks, mice were fed a HFD (60% fat and 20% carbohydrates (kcal per 100 g), D12492, (Research diet). For the last week of follow-up, a subset of mice was placed again in metabolic chambers to monitor individual food intake.

### Short-term HFD exposure

Two different cohorts of 10-weeks-old WT or *Napepld*^∆IEC^ male mice (67 mice in total, *n* = 16–17 per group) was housed individually during one week for acclimation in specific pathogen free conditions and in a controlled environment (room temperature of 23 ± 2 °C, 12 h daylight cycle) with free access to ND sterile food (irradiated) and sterile water. They were then split in four body weight-matched groups and either fasted for 20 h (fasted) or fasted for 16 h before exposure to a HFD for 4 h (HFD-Fed) before tissue harvesting.

### Fasting and refeeding experiment

An additional cohort of WT and *Napepld*^*ΔIEC*^ male mice (42 mice, *n* = 10 for WT groups and 11 for KO groups) were housed in pairs in specific pathogen-free conditions and in controlled environment (room temperature of 23 ± 2 °C, 12 h daylight cycle) with free access to sterile ND (irradiated) and sterile water until the day of final the experience. The animals were fasted overnight (ON) during the dark phase and refeed the following morning with ND or HFD for 1 h. Animals were anesthetized with IP injection of ketamine-xylazine (100 mg/ml and 20 mg/ml, respectively) and then transcardiacally perfused using first a solution of cold phosphate-buffered saline (PBS) and after cold 4% (w/v) paraformaldehyde (PFA). The entire brain was carefully harvested, post-fixed in 4% PFA ON at 4 °C, cryoprotected ON at 4 °C in a solution of sucrose 30% (w/v), subsequently frozen in cold iso-pentane and stored at −80 °C.

### Lipid load experiment

A new cohort of mice (42 mice, *n* = 10 for WT ND, *Napepld*^∆IEC^ ND and WT HFD groups and *n* = 12 for *Napepld*^∆IEC^ HFD) was intended to the study of lipid absorption after oral lipid challenge. An oral lipid dynamic test was performed after 7 weeks of HFD exposure. Overnight-fasted animals were gavaged with 300 μl of intralipid 20% emulsion (from Sigma); peripheral blood was sampled from the tail vein before and 1, 2, 3, 4, and 6 h after the Intralipid administration. Heparinated tubes were used for the sampling. Plasma was stored at −80 °C.

### Long-term phenotyping and assessment of energy expenditure

A cohort of 10-weeks-old WT or *Napepld*^∆IEC^ male mice (40 mice, *n* = 10 per group) was housed in pairs in specific pathogen-free conditions and in a controlled environment (room temperature of 23 ± 2 °C, 12 h daylight cycle) with free access to food and water. The mice were fed a ND (AIN93Mi; Research diet) or a HFD (60% fat and 20% carbohydrates (kcal per 100 g), D12492i, Research diet). Treatment continued for 16 weeks. Body weight, food and water intake were recorded weekly. Body composition (lean and fat mass) was assessed weekly by using 7.5 MHz time domain-nuclear magnetic resonance (TD-NMR; LF50 minispec, Bruker). After 15 weeks of follow-up, a subset of mice was placed in metabolic chambers for indirect calorimetry measurements.

### Fecal energy content

Fecal energy was measured on fecal samples harvested after a 24 h period during the final week of treatment by the use of a bomb calorimeter (Staufen, Germany).

### Treatment of Napepld^∆IEC^ mice with *Akkermansia muciniphila*

Data displayed in Supplementary Figure 3 come from a set of mice metabolically characterized in Everard et al.^36^. A cohort of 10 weeks-old WT or *Napepld*^∆IEC^ male mice (40 mice, *n* = 10 per group) was housed in pairs in specific pathogen free conditions and in a controlled environment (room temperature of 23 ± 2 °C, 12 h daylight cycle) with free access to food and water. The mice were fed a ND (AIN93Mi; Research diet) or a HFD (60% fat and 20% carbohydrates (kcal per 100 g), D12492i, Research diet) and treated daily with an oral gavage of either 2.10^8^ CFU of *Akkermansia muciniphila* in 150 µl sterile PBS containing 2.5% glycerol (culture conditions described below) or 150 µl of vehicle solution (PBS containing 2.5% glycerol). Body weight, food and water intake were recorded weekly. Body composition (lean and fat mass) was assessed weekly by using 7.5 MHz time domain-nuclear magnetic resonance (TD-NMR; LF50 minispec, Bruker). Treatment continued for 5 weeks. An oral glucose tolerance test was performed after 4 weeks of treatment.

### Oral glucose tolerance test

After 7 weeks of treatment, 6 h-fasted mice were given an oral glucose load (2 g glucose per kg body weight) and blood glucose levels were measured before and 15, 30, 60, 90, and 120 min after oral glucose load. Blood glucose was measured with a standard glucose meter (Accu Check, Roche, Basel, Switzerland) on the tip of the tail vein. Plasma samples were collected from the tip of the tail vein in heparinated tubes 30 min before and 15 min after oral glucose load for determination of insulin concentration.

### Insulin resistance index

Plasma insulin concentration was determined on samples using an ultrasensitive mouse insulin ELISA kit (Mercodia, Uppsala, Sweden) according to the manufacturer’s instructions. Insulin resistance index was determined by multiplying the area under the curve of both blood glucose (−30 to 120 min) and plasma insulin (−30 and 15 min) obtained following the oral glucose tolerance test.

### Insulin tolerance test

After 7 weeks of treatment, 6 h-fasted mice were intraperitoneally injected with insulin (0.5 U per kg body weight). Blood glucose levels were measured before and 15, 30, 45, 60, 90, and 120 min after insulin injection with a standard glucose meter (Accu Check, Roche, Basel, Switzerland) on the tip of the tail vein.

### Tissue sampling

At the end of the treatment period, mice were anesthetized with isoflurane (Forene, Abbott, Queenborough, Kent, England) after a fasting period of 6 h. Blood was sampled from the portal and cava veins. After exsanguination, mice were killed by cervical dislocation. Tissues were precisely dissected, weighed and immediately immersed in liquid nitrogen followed by storage at −80 °C for further analysis.

To analyze the insulin signaling pathway in the cohort subjected to the insulin tolerance test, mice were allocated to either a saline-injected subgroup or an insulin-injected subgroup so that both subgroups were matched in terms of body weight and fat mass. They then received 1 mU insulin/g body weight (Actrapid; Novo Nordisk A/S, Denmark) under anesthesia with isoflurane (Forene, Abbott, England), or an equal volume of saline solution into the portal vein. Three minutes after injection, mice were killed and liver was harvested.

### Culture of *Akkermansia muciniphila*

*A. muciniphila* MucT (ATTC BAA-835) was cultured anaerobically in a basal mucin medium^47^ where mucin was replaced by 16 g/l soy-peptone, 4 g/l threonine, and a mix of glucose and *N*-acetylglucosamine (25 mM each)^37^. Cultures were washed and concentrated in anaerobic PBS with 25% (vol/vol) glycerol under strict anaerobic conditions. Cultures were then immediately frozen and stored at −80 °C. A representative glycerol stock was thawed under anaerobic conditions to determine the CFU/ml by plate counting using mucin-supplemented BHI medium containing 1% agarose (agar noble, Difco). Before administration by oral gavage, glycerol stocks were thawed under anaerobic conditions and diluted with anaerobic PBS to an end concentration of 2 × 10^8^ CFU/150 µl and 2.5% glycerol.

### RNA preparation and real-time qPCR analysis

Total RNA was prepared from tissues using TriPure reagent (Roche). Quantification and integrity analysis of total RNA were performed by analyzing 1 μl of each sample in an Agilent 2100 Bioanalyzer (Agilent RNA 6000 Nano Kit, Agilent, Santa Clara, California, USA). cDNA was prepared by reverse transcription of 1 μg total RNA using a Reverse Transcription System kit (Promega, Madison, Wisconsin, USA). Real-time PCR was performed with the CFX96 real-time PCR system and CFX Manager 3.1 software (Bio-Rad, Hercules, California, USA) using Mesa Fast qPCR (Eurogentec, Liège, Belgium) for detection according to the manufacturer’s instructions. *Rpl19* RNA was chosen as the housekeeping gene. All samples were performed in duplicate, and data were analyzed according to the 2^−ΔΔCT^ method. The identity and purity of the amplified product were assessed by melting curve analysis at the end of amplification. The primer sequences for the targeted mouse genes are presented in Supplementary Table 3.

### Western-blot analyses

For the detection of NAPE-PLD, tissues were homogenized with TissueLyser II (Qiagen) in RIPA buffer supplemented with a cocktail of protease inhibitors and phosphatase inhibitors (Sigma). Equal amounts of proteins were separated by SDS–PAGE and transferred to nitrocellulose membranes. Membranes were incubated overnight at 4 °C with antibodies against NAPE-PLD and β-actin diluted in Tris-buffered saline tween-20 containing 1% non-fat dry milk.

For detection of Akt, AMPK and their phosphorylated forms, tissues were homogenized in ERK buffer (Triton X-100 0.1%, HEPES 50 mM, NaCl 5 M, Glycerol 10%, MgCl_2_ 1.5 mM and DTT 1 mM) supplemented with a cocktail of protease inhibitors and phosphatase inhibitors. Equal amounts of proteins were separated by SDS–PAGE and transferred to nitrocellulose membranes. Membranes were incubated overnight at 4 °C with antibodies against total Akt, p-Akt^Thr308^ and p-Akt^Ser473^ or AMPK and p-AMPK^Thr172^ diluted in Tris-buffered saline tween-20 containing 1% non-fat dry milk or 1% bovine serum albumin.

The revelation was performed using a chemiluminescent substrate (SuperSignal^®^ West Pico (ThermoScientific) and Amersham Image600 (GE Healthcare). Densitometry analysis were performed using ImageQuanTL software.

Information regarding all the antibodies can be found in Supplementary table 4.

Uncropped western blots are available as Supplementary Figures 7–9.

### Histology on brain

Twenty micrometer thick serial coronal cryosection were mounted on SuperFrost Plus slides (Menzel Gläser) and kept at −20 °C. For Arcuate Nucleus we harvested approximately 10 serial sections per animal from bregma −1.22 mm to −2.54 mm according to The Mouse Brain in stereotaxic coordinates (Paxinos, Franklin); for the Paraventricular Nucleus 6 sections per animal from bregma −0.58 mm to −1.22 mm.

For c-Fos immunohistochemistry in PVN, after antigen retrieval (Dako S1699) by heating (2100 Antigen Retriever, from Aptum) the endogenous peroxidases were inhibited in a solution of MeOH with H_2_O_2_ 0.1% (v/v)_._ The sections were incubated for 45 min at RT in blocking solution (TBS, BSA 5%, Tween20 0.1%) and then incubated ON at RT with a primary anti-c-Fos (1/2000, ab190289 from Abcam). After washing, sections were incubated for 1 h at RT with secondary antibody (Envision Kit, Dako K4010); the diaminobenzidine-mediated chromogenic detection was developed following manufacturer’s instruction (Envision kit, Dako K4010). Slides were dehydrated and covered.

Bright field images of the PVN were obtained using Leica scanner (Leica SCN400). After blinding procedure, using Fiji software^48^ the region of interest (ROI) corresponding to the PVN was delimited on each section using The Mouse Brain in stereotaxic coordinates (Paxinos, Franklin) as reference. c-Fos-positive neurons were manually counted within each ROI and a mean value was obtained for each animal (considering at least three brain sections per animal).

Double immunofluorescence c-Fos/POMC in ARC was performed using Tyramide-signal amplification (TSA) technology. After antigen retrieval, inhibition of endogenous peroxidases and blocking (as described above), the sections were incubated ON with rabbit anti-c-Fos (1/10,000, ab190289 from Abcam). Sections were incubated 1 h at RT with HRP-conjugated secondary antibody (DAKO K4003). The fluorescent signal was amplified using TSA Cyanine 5 system (TSA Plus Fluorescence Kit, PerkinElmer) according to manufacturer’s protocol. According to the TSA procedure, the primary and the secondary antibodies were removed by heat-treatment in the antigen retrieval solution. This step does not affect the fluorescent signal^49^. After blocking, sections were incubated with rabbit anti-POMC primary antibody (1/1000, ab94446 from Abcam), then HRP-conjugated secondary antibody (DAKO K4003). Staining was developed by FITC-tyramide (TSA Fluorescence System, PerkinElmer) according to the manufacturer’s instructions. Finally, all the sections were counterstained with Hoechst33342 and auto-fluoresce was inhibited using TrueBlack (from Biotium). Considering that both the primary antibodies are produced in rabbit, all the appropriate control were performed in order to verify the lack of cross-reactivity. More in detail, after incubation of the brain sections with the primary rabbit-anti-c-Fos, the fluorescent signal was developed according to the protocol described above using Cyanine 5-tyramide. After the heat-treatment the section were incubated with secondary antibody anti-rabbit and a FITC-tyramide. No FITC signal was observed on the sections.

c-Fos and POMC quantification in ARC were obtained using Pannoramic P250 slide scanner. c-Fos and POMC-expressing neurons were quantified on multiplex-stained cryosections with software applications (APP“s) using the image analysis tool Author version 2017.2 (Visiopharm, Hørsholm, Denmark). Brightfield (blue counterstaining of tissue) and fluorescent (c-Fos/POMC/Hoechst33342) scans were first aligned using the Tissuealign add-on from Visiopharm. Regions of interest (ROI) were then manually delineated on the brightfield scans and were automatically applied to the aligned fluorescent scans. Within these ROIs, nuclei were segmented based on the Hoechst nuclear staining and classified into four categories according to their expression of c-Fos and POMC (negative, single stained for each marker, double stained). Detected cells were finally quantified using a counting frame to avoid double cell counting. The same APP was applied to all the sections. Results were expressed as number of double stained neurons c-Fos/POMC over the the number of total c-Fos positive neurons, within a ROI. For each animal an average value was obtained.

### Quantification of intestinal eCB and eCB-like levels

Data presented in Fig. 1 and corresponding to intestinal epithelial cells were obtained as follows: intestinal epithelial cells isolated as described in the ref. ^35^ and were homogenized in CHCl_3_ (10 ml), and a deuterated standard (200 pmol) was added. Methanol (5 ml) and H_2_O (2.5 ml) were added, and the lipids were then extracted by vigorous mixing. After centrifugation, the organic layer was recovered, dried under a stream of N_2_ and purified by solid-phase extraction using silica, followed by elution with an EtOAc-Acetone (1:1) solution. The resulting lipid fraction was analysed by high-performance liquid chromatography-MS using an LTQ Orbitrap mass spectrometer (ThermoFisher Scientific) coupled to an Accela HPLC system (ThermoFisher Scientific). Analyte separation was achieved by using a C-18 Kinetex C-18 column (5 mm, 4.6 × 150 mm; Phenomenex) and a C18 pre-column. Mobile phases A and B were composed of MeOH/H_2_O/acetic acid 75:25:0.1 (v/v/v) and MeOH/acetic acid 100:0.1 (v/v), respectively. The gradient (0.5 ml min^−1^) was designed as follows: transition from 100% A to 100% B linearly over 15 min, followed by 10 min at 100% B and subsequent re-equilibration at 100% A. We performed mass spectrometry analysis in the positive mode with an APCI ionization source. The capillary and APCI vaporizer temperatures were set at 250 and 400 °C, respectively. The eCBs were quantified by isotope dilution using their respective deuterated standard (showing identical retention times). The calibration curves were generated as described and the data were normalized to the average eCB content/tissue weight of the WT group^3^.

### LC-MS/MS analyses of endocannabinoids

Data displayed in Figs. 3,  10 and Supplementary Figure 3 were obtained as follows: intestinal epithelial cells Frozen (−196 °C) jejunum samples (~10 mg) were crushed with a disposable tissue grinder, harvested with 500 µl PBS, immediately mixed with 500 µl methanol containing 5 ng of deuterated standards (see table), vortexed for 60 s then agitated 90 min at room temperature to fully denature the proteins. Samples were then centrifuged (20,000×*g*; 10 min, room temperature). The supernatants were collected and their methanol content adjusted to 10% with acidified water (0.05 acetic acid). Lipids were next extracted from the samples using solid phase extraction cartridges (Strata-X Polymeric Reversed Phase, 60 mg/1 ml, Phenomenex). In brief, cartridges were washed with 2 ml acidified methanol (0.05% acetic acid), then with 2 ml acidified water (0.05% acetic acid). Samples were loaded on cartridges, cartridges were washed with 2 ml acidified water, and lipids were eluted with 1 ml acidified methanol. The eluates were dried under a stream of nitrogen and reconstituted in 50 µl of the mobile phase (50% B). Recoveries of the different compounds averaged 50% ± 7% (mean ± sem), 2-palmitoyl-glycerol being the lowest (35.2 ± 4.9) and EPA-2-glycerol being the highest (71.5 ± 4.5) 40 µl of the samples were injected onto an HPLC column (Kinetex C8, 150 × 2.1 mm, 2.6 μm, Phenomenex) and eluted at a flow rate of 400 μl/min using a discontinuous gradient of solvent A (1 mM ammonium acetate + 0,05% acetic acid) and solvent B (acetonitrile/water; 95/5 + 1 mM ammonium acetate + 0.05 acetic acid). Gradient was as follows: 15–35% B from 0 to 2 min, 35–75% B from 2 to 12 min, 75–95% B from 12 to 12.1 min and kept at 95% until 17 min. The HPLC system was interfaced with the electrospray source of a Shimadzu 8050 triple quadrupole mass spectrometer and mass spectrometric analysis was done in the positive ion mode using multiple reaction monitoring using the specific mass transitions shown in Supplementary Table 11.

### Lipidomics analysis in the jejunum

Data displayed in supplementary tables 5–10 were obtained by using lipidomics performed in collaboration with Biocrates (Innsbruck, Austria). The biologically most abundant members of (lyso-) glycerophospholipids, i.e., (lyso-) glycerol-phosphocholines, glycerol-ethanolamines, glycerol-serines, glycerol-glycerols, as well as sphingolipids, i.e., sphingomyelins, ceramides, dihydroceramides, and 2-hydroxyacyl ceramides, were quantitatively analyzed by a high-throughput flow injection ESI-MS/MS screening method. The MRM detection in positive and negative mode was performed using a AB SCIEX 4000 QTrap® tandem mass spectrometry instrument (AB SCIEX, Darmstadt, Germany). The sample preparation of 20 μl sample volume followed a MeOH/CHCl3-liquid/liquid-extraction protocol. Five internal standards were used to compensate for matrix effects, and 43 external standards for a multipoint calibration. The quantitative data analysis was performed with Biocrates in-house software MetIDQ™ enabling isotopic correction.

### Extraction of liver lipids

Total lipids were measured in the liver tissue after extraction in CHCl3:MeOH according to^50^ and adapted as follows: briefly, 100 mg of liver tissue was homogenized in 2 ml of CHCl_3_:MeOH (2:1) using a Tissue Lyser followed by an ultrasonic homogenizer. Four hundred microliter of 0.9% NaCl solution was added and lipids were then extracted by vigorous shaking. After centrifugation, the chloroform phase was recovered in glass tubes and dried under a stream of N_2_. Glass tubes were weighed before and after lipid extraction to quantify total lipid content. The dried residue was solubilized in 1.5–3 ml isopropanol depending on the lipid content.

### Biochemical analyses

Plasma non-esterified fatty acids (NEFA) and liver and plasma cholesterol and triglyceride concentrations were measured using kits coupling an enzymatic reaction with spectrophotometric detection of the reaction end-products (Diasys Diagnostic and Systems, Holzheim, Germany) according to the manufacturer’s instructions.

Plasma alanine-aminostransferase (ALT) and aspartate-aminotransferase (AST) activities were measured using kits coupling the enzymatic activity of interest with a NADH+H+-dependent enzymatic reaction and spectrophotometric detection of NADH+H+conversion to NAD+(Diasys Diagnostic Systems, Holzheim, Germany) according to the manufacturer’s instructions.

Portal GLP-1 and ghrelin were determined in duplicate using a Bio-Plex Pro Assays kit (Bio-Rad, Nazareth, Belgium) and measured using Luminex (Bio-Rad Bioplex; Bio-Rad) according to the manufacturer’s instructions. Liver oxidative stress level was evaluated by measuring lipid peroxidation and reactive compounds such as malondialdehyde (MDA) and 4-hydroxynonenal, natural byproducts of lipid peroxidation. Briefly, 50 mg of tissue were homogenized in cold 0.9% NaCl at 100 mg tissue/ml. Three aliquots of 100 µl homogenate were used for the measurements. Seven hundred and fifty microliter of H3PO4 1% were added to each homogenate. Two hundred and fifty microliter TBA 0.6% were added to two out of three homogenates, while 250 µl water was added the third for blank measurements. Samples and blanks were incubated 1 h at 95 °C then put on ice. 12.5 µl HCl 1 N and 1 ml *N*-butanol were added to each tube and lipids were then extracted by vigorous mixing followed by centrifugation 20 min at 3000 × *g* and 4 °C. Adducts formed in samples due to the reaction between MDA with thiobarbituric acid were measured spectrophotometrically using a Spectramax M2 instrument (Molecular Devices) according to the manufacturer’s instructions. TBARS levels were determined from a MDA equivalence standard. Protein concentrations were measured by the Bradford method using bovine serum albumin as a standard.

### DNA isolation from mouse cecal samples for sequencing

Cecal contents were collected and kept frozen at −80 °C until use. Metagenomic DNA was extracted from the cecal content using a QIAamp DNA Stool Mini Kit (Qiagen, Hilden, Germany) according to the manufacturer’s instructions with modifications^34^.

### Bacterial DNA sequencing

The V1–V3 region of the bacterial 16S rRNA gene was amplified using barcoded primers 27f (5′-CCTATCCCCTGTGTGCCTTGGCAGTCTCAG-3′) and 534r (5′-ATTACCGCGGCTGCTGG-3′) and high-throughput sequencing of purified amplicons were analysed on a Roche FLX Genome Sequencer using the Titanium chemistry (DNAVision). Resulting reads were processed through QIIME v1.9.0 pipeline. OTUs were identified using the uclust consensus taxonomy classifier with a 0.97 threshold against the Greengenes database (Fig. 5c–f) or using mother (Supplementary Tables 1–2 and Supplementary Data 1). Principal coordinate analysis was calculated using unweighted UniFrac distance. Phylogenetic tree was generated using QIIME 1.9.0 and visualized using GraPhIAn^51^. Sequence data that support the findings of this study have been deposited in Sequence Read Archive (SRA) database with the PRJNA508476 accession code (http://www.ncbi.nlm.nih.gov/sra/PRJNA508476).

### Hepatic lipid content analysis by Oil Red O staining

Liver tissue was embedded in Tissue-Tek Optimal Cutting Temperature compound (Sakura Europe, Leiden, Netherlands) and flash-frozen in cold isopentane. Five micrometer-tick tissue sections were stained with Oil red O staining for lipid content analysis. Five high-magnification fields (20×) were analyzed per mouse. Quantification of mean droplet area was performed using ImageJ software (Version 1.50a, National Institutes of Health, Bethesda, Maryland, USA).

### Fibrosis analysis by Sirius red staining

Hepatic collagen content was visualized by staining paraffin sections with 0.1% Sirius Red (Sigma-Aldrich). At least five high-magnification fields were selected at random for each mouse. Images were obtained using a SCN400 slide scanner and Digital Image Hub software (Leica Biosystems, Wetzlar, Germany). All analyses were performed in a blinded manner by the investigator and quantified using an semi-automated script in ImageJ software (Version 1.50a, National Institutes of Health, Bethesda, Maryland, USA). Collagen content was quantified as percentage stained area per total tissue section area.

### Statistical analysis

Statistical analyses were performed using GraphPad Prism version 7.00 for Windows (GraphPad Software, San Diego, CA, USA) except for microbiota analyses as described above. If variances were significantly different between groups, values were normalized by log-transformation before proceeding to the analysis. In cases when variance differed significantly between groups even after normalization, a non-parametric test was performed. Different with *P* values < 0.05 were considered significant.

### Reporting Summary

Further information on experimental design is available in the Nature Research Reporting Summary linked to this Article.

## Supplementary Information

Supplementary Information

Description of Additional Supplementary Files

Supplementary Data1

Reporting Summary

## Data Availability

All lipidomic data are included in this Article as Supplementary Information (Supplementary Tables 5–10). All the remaining data will be made available by the corresponding author upon reasonable request.
