# Supplementary material for: Intestinal epithelial N-acylphosphatidylethanolamine phospholipase D links dietary fat to metabolic adaptations in obesity and steatosis
Source: Nat Commun. 2019 Jan 28;10:457. doi: 10.1038/s41467-018-08051-7 (PMC6349942; doi:10.1038/s41467-018-08051-7)
Supplement: Supplementary file 1 — Supplementary Information [file 41467_2018_8051_MOESM1_ESM.pdf]

Supplementary information

Intestinal epithelial N-acylphosphatidylethanolamine phospholipase D links dietary fat to metabolic adaptations in obesity and steatosis

Everard, Plovier, Rastelli et al.

Supplementary Figures

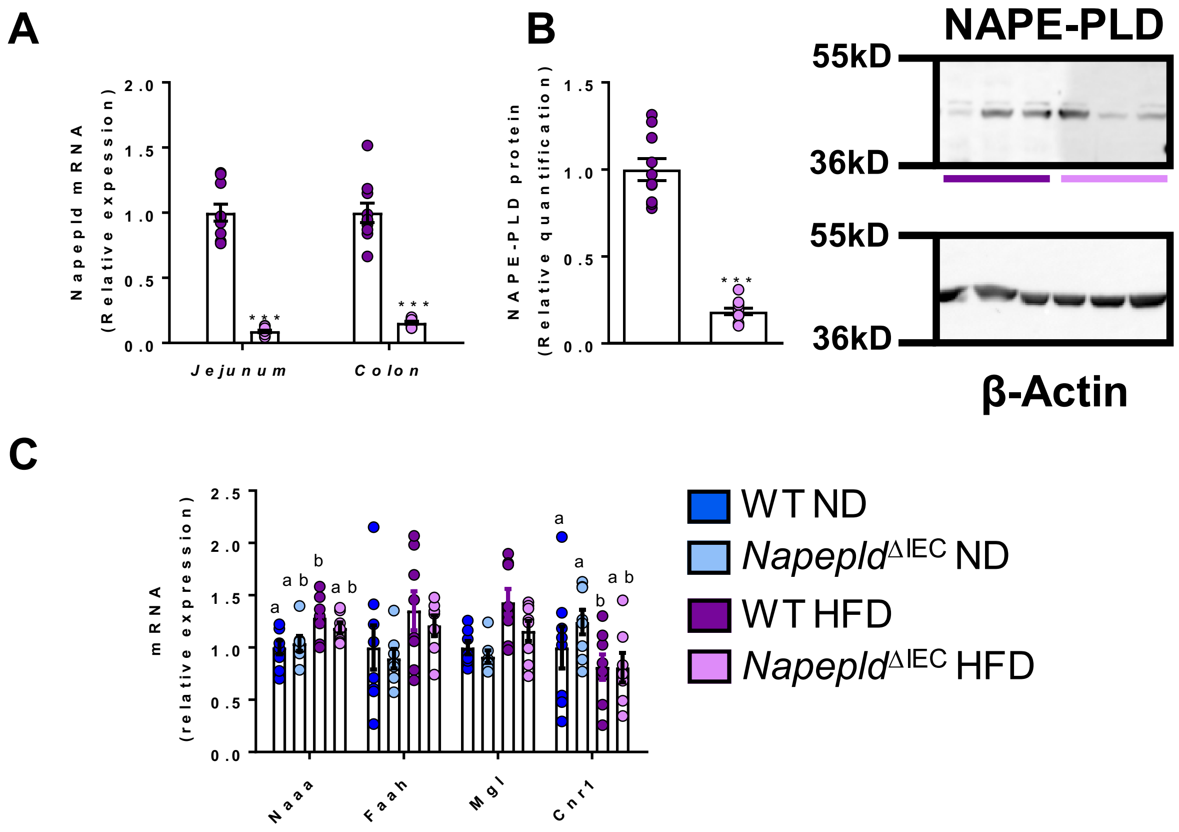

Supplementary Figure 1: Validation of IEC *Napepld* deletion under HFD. Related to Figure 1. A) *Napepld* mRNA expression in the jejunum, colon in HFD-fed WT and *Napepld*$^{\Delta IEC}$ mice (n = 8-10). B) NAPE-PLD protein levels in the colon. Representative western-blot of NAPE-PLD and β-Actin (n = 9-10). Relative mRNA expression of *NAAA, Faah, Mgl,* and *Cnr1* in the ileum of WT and *Napepld*$^{\Delta IEC}$ mice either fed with ND or HFD for 8weeks (n = 8). Data are presented as the mean ± s.e.m. '***' indicates a significant difference versus WT HFD (P<0.001) according to Mann-Whitney test. Data with different superscript letters are significantly different (P<0.05) according to regular two-way ANOVA followed by Tukey's post-hoc test.

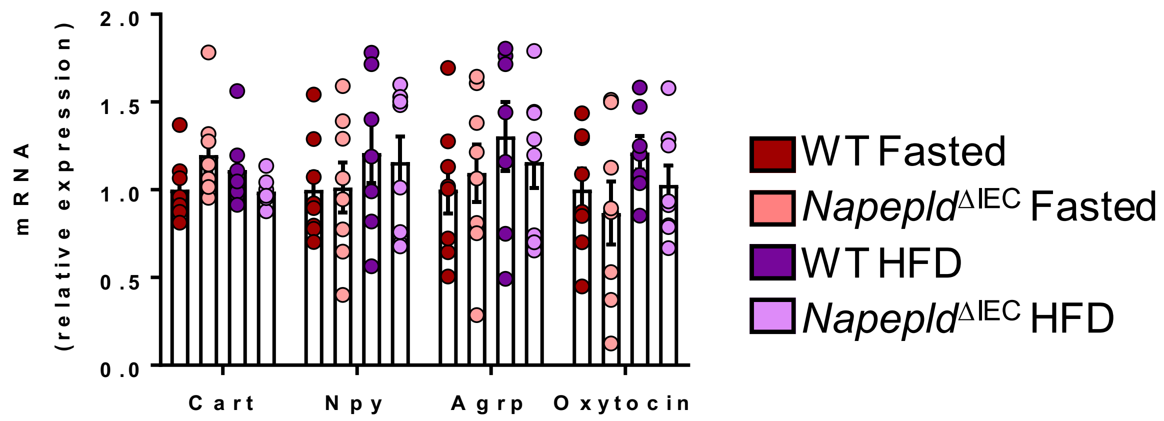

Supplementary Figure 2: Consequences of IEC *Napepld* deletion on the expression of genes regulating food intake. Related to Figure 3. mRNA expression of genes (*Cart*, *Npy*, *Agrp*, *Oxytocin*) involved in the regulation of food intake in the hypothalamus (n = 7-8). Data are presented as the mean ± s.e.m.

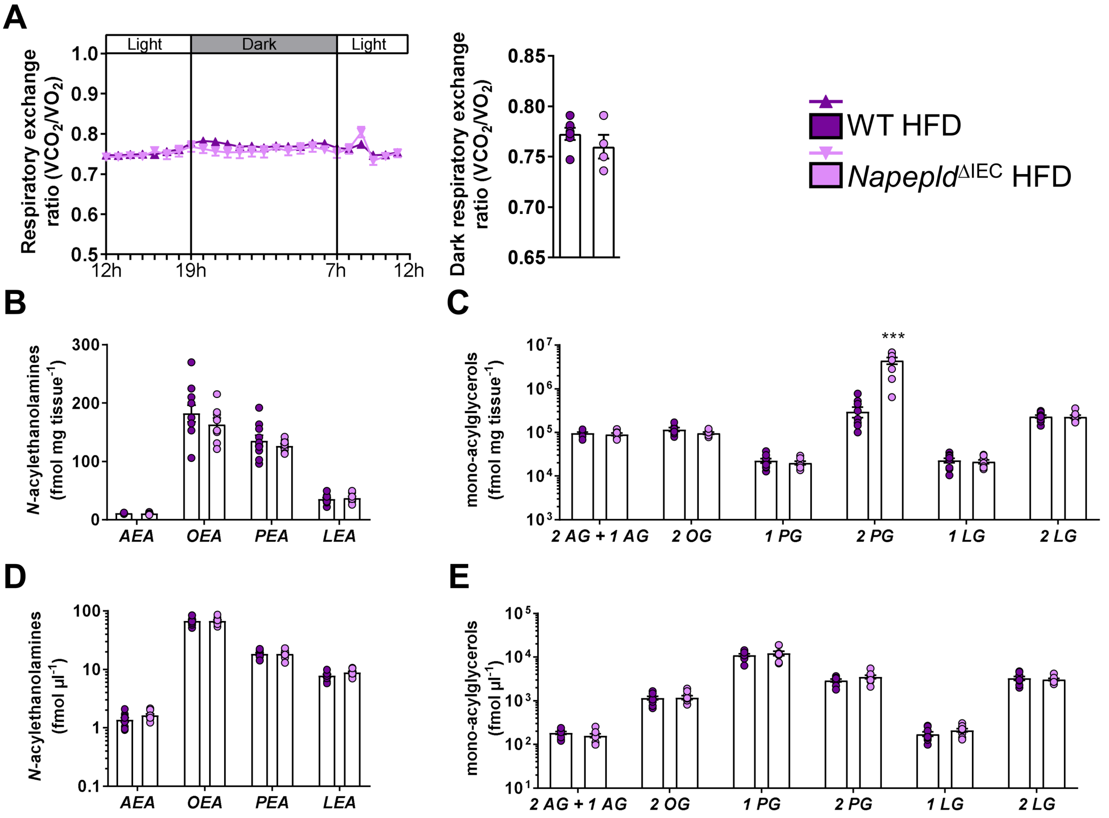

Supplementary Figure 3: Respiratory exchange ratio and intestinal and portal plasma *N*-acylethanolamines and mono-acylglycerols at the end of 16weeks HFD. Related to Figure 5. A) Light and dark cycle of respiratory exchange ratio (VCO_2/VO_2) (n = 4-6). B) *N*-acylethanolamines (fmol mg tissue^{-1}) measured in the jejunum (n=8-10). C) mono-acylglycerols (AG= 1AG+2AG) (fmol mg tissue $^{-1}$) measured in the jejunum (n=8-10). D) *N*-acylethanolamines (fmol µl^{-1}) measured in the portal vein (n=9-10). J) mono-acylglycerols (AG= 1AG+2AG) (fmol µl^{-1}) measured in the portal vein (n=8-10). Data are presented as the mean ± s.e.m. '***' indicate a significant difference versus WT HFD (P<0.001) according to Mann-Whitney test.

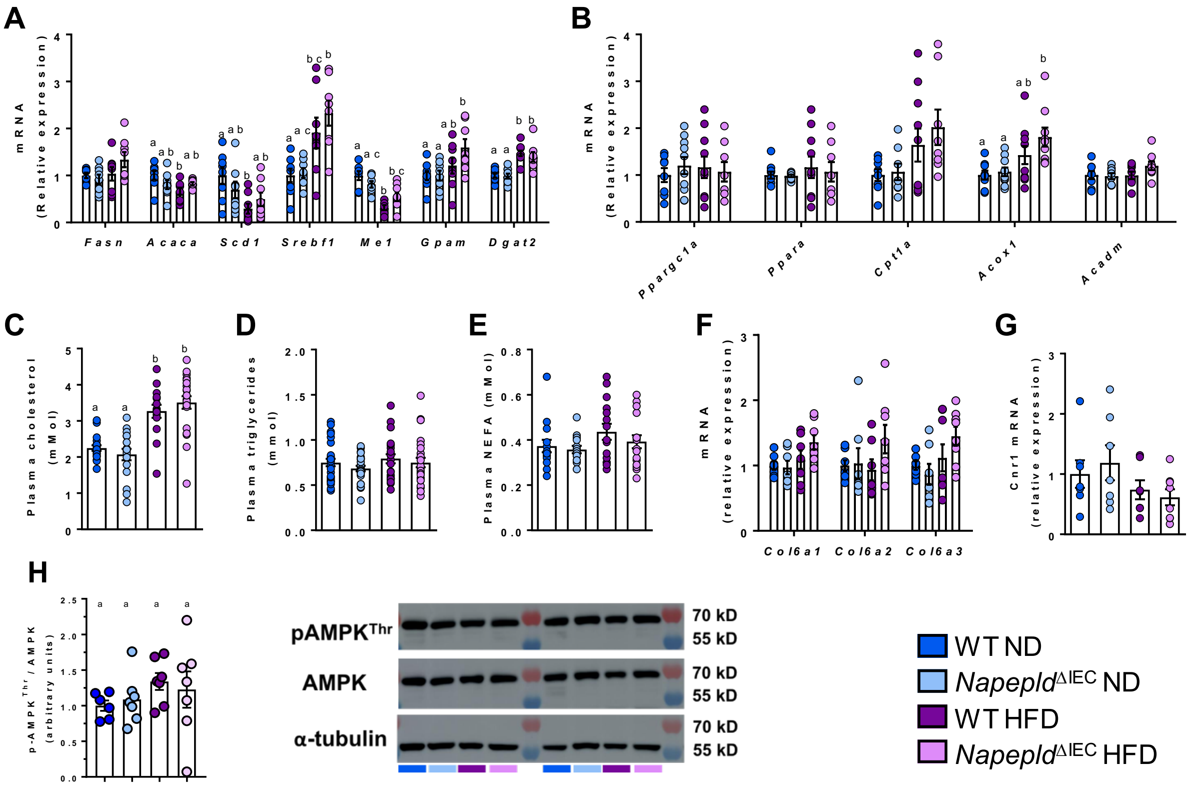

Supplementary Figure 4: Effects of IEC *Napepld* deletion on liver gene expression and on circulating lipids. Related to Figure 6. A) mRNA expression of genes involved in fatty acid synthesis in the liver (n = 7-8). B) mRNA expression of genes involved in fatty acid oxidation in the liver (n = 8). C) Plasma cholesterol levels (mMol, n = 15-21). D) Plasma triglycerides levels (mMol, n = 26-28). E) Plasma non-esterified fatty acids levels (mMol, n = 14-16). F) mRNA expression of genes encoding collagen fibers in the liver (n = 7-8). G) mRNA expression of *Cnr1* in the liver (n = 7-8). H) Representative western-blot of AMPK (62 kDa) , p-AMPK ^{Thr172} (62 kDa) and α-tubulin (50 kDa) (n = 6-7). Data are presented as the mean ± s.e.m. Data with different superscript letters are significantly different (P<0.05) according to regular two-way ANOVA followed by Tukey's post-hoc test.

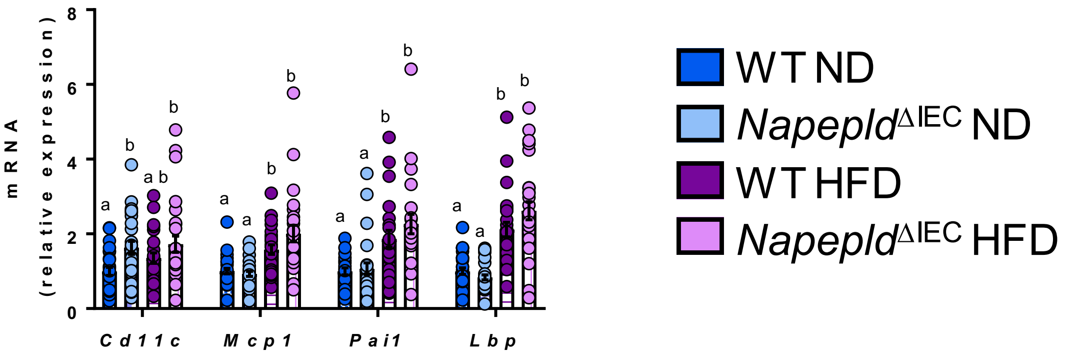

Supplementary Figure 5: Effects of IEC *Napepld* deletion on adipose tissue inflammation. mRNA expression of inflammatory markers in the visceral adipose tissue (n = 25-27). Data are presented as the mean $\pm$ s.e.m. Data with different superscript letters are significantly different (P<0.05) according to regular two-way ANOVA followed by Tukey's post-hoc test.

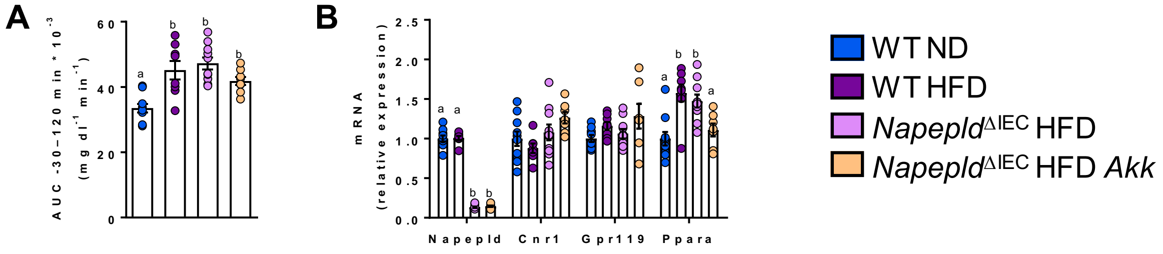

Supplementary Figure 6: Effects of *A. muciniphila* on glucose tolerance and gene expression related to the NAE in HFD-fed *Napepld*$^{\Delta IEC}$ mice. Related to Figure 9. A) Mean area under the curve (AUC, mg dl^{-1} min^{-1} 10^{-3}) measured between 30 minutes before and 120 min after glucose loading (n=8-10). B) mRNA expression of *Napepld*, Cnr*1*, *Gpr119* and *Ppara* in the ileum (n=7-10). Data are presented as the mean $\pm$ s.e.m. Data with different superscript letters are significantly different (P<0.05) according to regular two-way ANOVA followed by Tukey's post-hoc test.

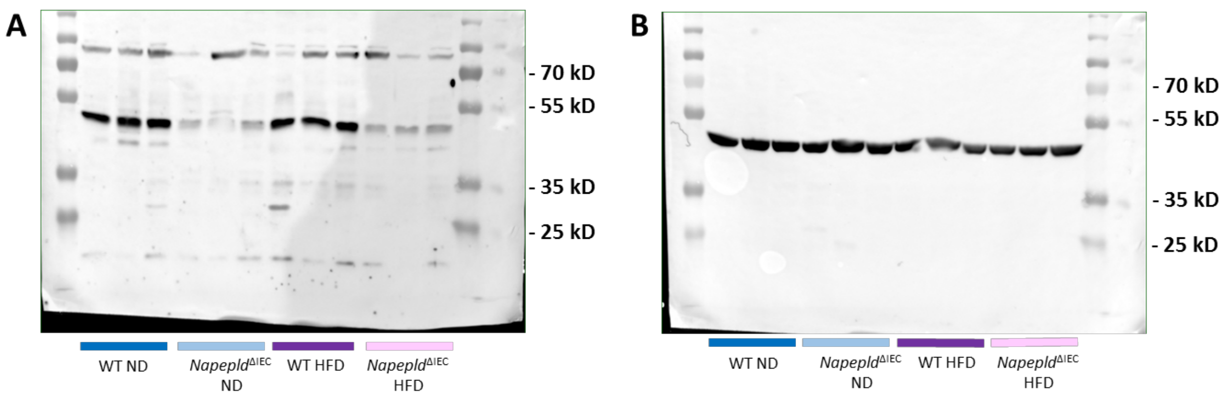

Supplementary Figure 7: Uncropped scans of the western blot presented in Fig. 1B and in Supplementary Figure 1. (**A**) NAPE-PLD and (**B**) β-actin.

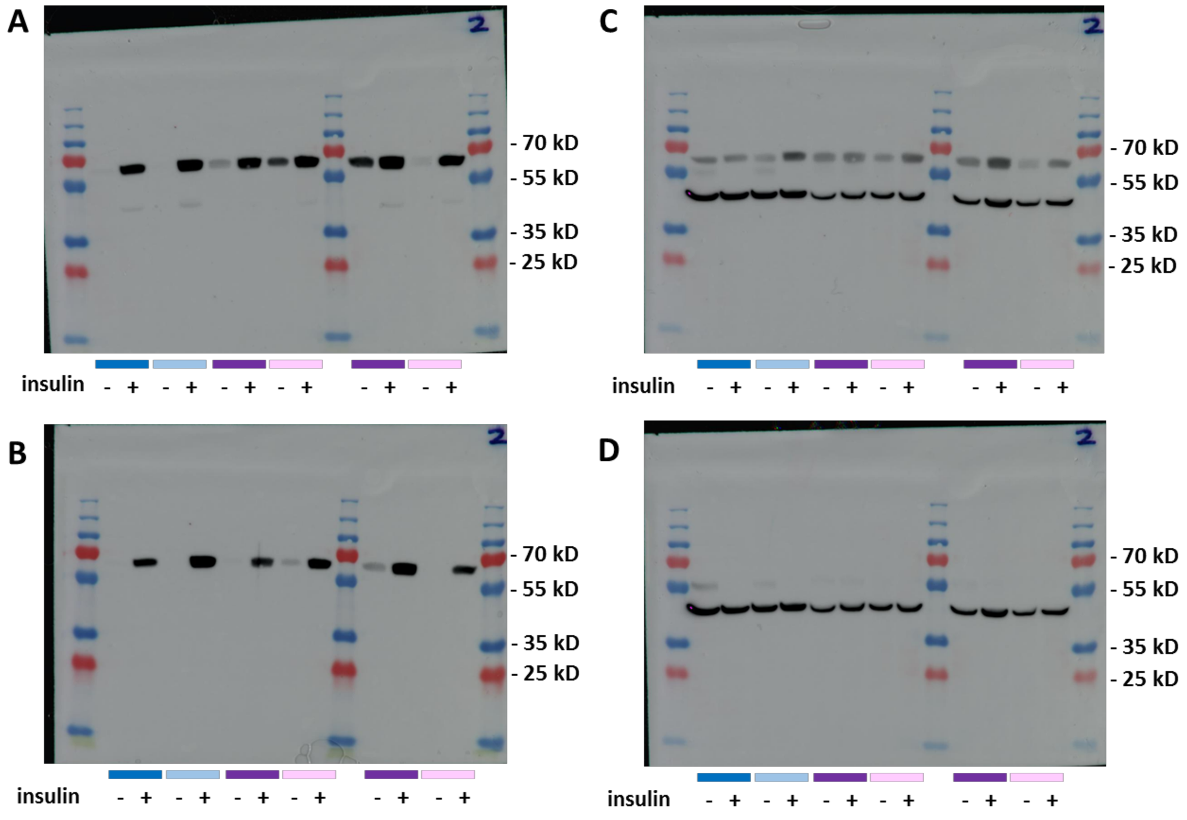

Supplementary Figure 8: Uncropped scans of the western blot presented in Fig. 7. **(A)** p-AKT ^{Ser473}, **(B)** p-AKT^{Thr308}, **(C)** non phosphorylated AKT; the band at 42 kD on blot C corresponds to the remaining of β-actin which was detected previously to AKT; **(D)** β-actin.

WT ND in dark blue , *Napepld*$^{\Delta IEC}$ ND in light blue, WT HFD in purple, *Napepld*$^{\Delta IEC}$ HFD in pink.

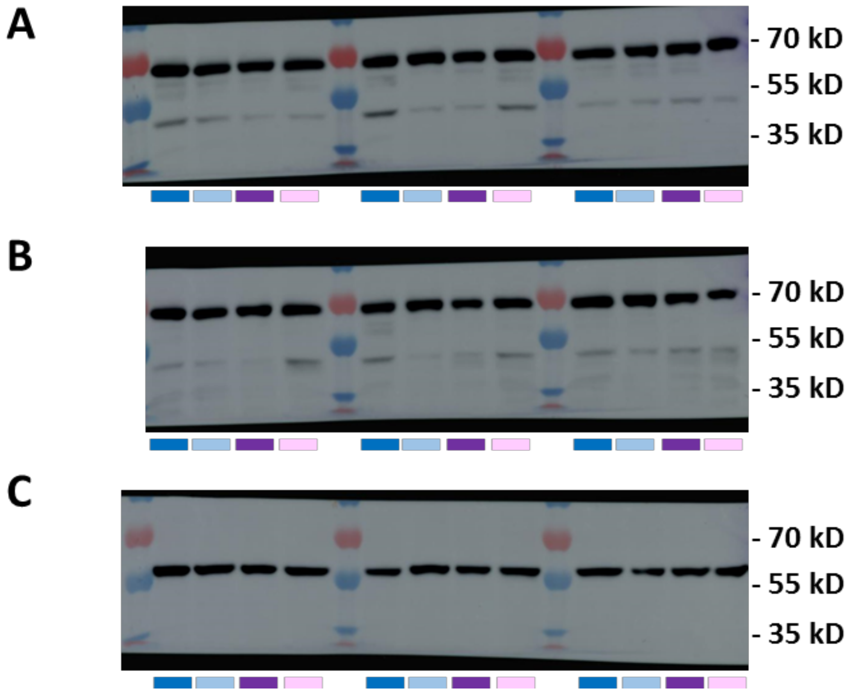

Supplementary Figure 9: Uncropped scans of the western blot in Supplementary Figure 4. **(A)** p-AMPK^{Thr172} , **(B)** non phosphorylated AMPK and **(C)** α-tubulin . The blot is cut at 100kDa.
WT ND in dark blu , *Napepld*$^{\Delta IEC}$ ND in light blue, WT HFD in purple, *Napepld*$^{\Delta IEC}$ HFD in pink.

Supplementary Table 1: Relative abundance of the bacterial phyla found in the different groups (% of 16S rRNA sequences), Related to Figure 5

	WT ND		*Napepld*$^{\Delta IEC}$ ND			
	Mean	SEM	Mean	SEM	Changes (%)	p-value
**Actinobacteria**	0,04	0,02	0,03	0,01	-15,77	0,7835
**Bacteroidetes**	29,91	2,49	35,56	2,27	18,89	0,1113
**Cyanobacteria**	0,11	0,03	0,10	0,04	-5,48	0,9162
**Deferribacteres**	4,53	1,06	4,20	0,79	-7,14	0,8068
**Firmicutes**	56,83	1,89	50,39	2,41	-11,33	0,0543
**kTM7**	0,05	0,02	0,01	0,00	-89,20	0,0539
**Proteobacteria**	5,90	0,38	7,59	0,51	28,53	0,0183
**Tenericutes**	1,48	0,56	1,13	0,28	-23,42	0,5791
**Unassigned**	1,16	0,13	0,99	0,09	-14,83	0,2852

	WT ND		WT HFD			
	Mean	SEM	Mean	SEM	Changes (%)	p-value
**Actinobacteria**	0,04	0,02	0,01	0,00	-82,16	0,1056
**Bacteroidetes**	29,91	2,49	30,54	1,22	2,12	0,8165
**Cyanobacteria**	0,11	0,03	0,05	0,02	-52,02	0,1302
**Deferribacteres**	4,53	1,06	1,74	0,27	-61,68	0,0154
**Firmicutes**	56,83	1,89	58,71	1,12	3,31	0,3930
**kTM7**	0,05	0,02	0,00	0,00	WT ND	0,0309
**Proteobacteria**	5,90	0,38	8,05	0,32	36,32	0,0004*
**Tenericutes**	1,48	0,56	0,10	0,03	-93,05	0,0196
**Unassigned**	1,16	0,13	0,80	0,09	-30,76	0,0401

	WT HFD		*Napepld*$^{\Delta IEC}$ HFD			
	Mean	SEM	Mean	SEM	Changes (%)	p-value
**Actinobacteria**	0,01	0,00	0,01	0,00	-14,27	0,85725
**Bacteroidetes**	30,54	1,22	26,64	1,93	-12,77	0,10546
**Cyanobacteria**	0,05	0,02	0,03	0,02	-42,87	0,42838
**Deferribacteres**	1,74	0,27	2,48	0,47	43,04	0,18467
**Firmicutes**	58,71	1,12	61,77	2,15	5,21	0,22312
**kTM7**	0,00	0,00	0,01	0,01	*Napepld*$^{\Delta IEC}$ HFD	0,10287
**Proteobacteria**	8,05	0,32	8,17	0,49	1,57	0,83047
**Tenericutes**	0,10	0,03	0,08	0,04	-19,32	0,67611
**Unassigned**	0,80	0,09	0,80	0,08	0,02	0,99900

All phyla detected by pyrosequencing are presented in the tables. Indication WT ND or *Napepld*$^{\Delta IEC}$ HFD in the column 'changes (%)' corresponds to the group of mice for which the given phylotype was found exclusively. P-values are based on Student *t*-test. u=unclassified. Significant P-values (≤0.05 are indicated in red). *: significant p-values after FDR correction.

Supplementary Table 2: Relative abundance of the bacterial families found in the different groups (% of 16S rRNA sequences), Related to Figure 5

	WT ND		*Napepld*$^{\Delta IEC}$ ND			
	Mean	SEM	Mean	SEM	Changes (%)	p-value
Alcaligenaceae	0,439	0,127	1,064	0,562	142,65	0,3168
Anaeroplasmataceae	1,299	0,558	0,669	0,243	-48,47	0,2981
Bacteroidaceae	0,922	0,128	1,178	0,157	27,72	0,2309
Caulobacteraceae	0,001	0,001	0,000	0,000	WT ND	0,3051
Christensenellaceae	0,090	0,021	0,050	0,011	-44,87	0,0915
Clostridiaceae	0,016	0,008	0,041	0,022	159,96	0,3225
Comamonadaceae	0,000	0,000	0,005	0,005	*Napepld*$^{\Delta IEC}$ ND	0,3574
Coriobacteriaceae	0,038	0,019	0,032	0,011	-15,77	0,7835
Deferribacteraceae	4,528	1,056	4,205	0,791	-7,14	0,8068
Dehalobacteriaceae	0,299	0,028	0,303	0,029	1,52	0,9115
Desulfovibrionaceae	4,926	0,285	5,333	0,560	8,27	0,5394
Enterobacteriaceae	0,002	0,002	0,003	0,003	11,18	0,9418
Erysipelotrichaceae	2,529	0,774	2,756	0,612	8,99	0,8189
Eubacteriaceae	0,001	0,001	0,000	0,000	WT ND	0,3051
F16	0,048	0,022	0,005	0,003	-89,20	0,0539
Lachnospiraceae	2,522	0,298	2,465	0,267	-2,27	0,8878
Lactobacillaceae	0,088	0,029	0,101	0,039	14,13	0,8018
Mogibacteriaceae	0,107	0,020	0,073	0,016	-31,52	0,1975
Odoribacteraceae	3,870	0,234	3,604	0,297	-6,87	0,4976
Peptococcaceae	0,196	0,039	0,201	0,032	2,83	0,9137
Porphyromonadaceae	0,192	0,050	0,339	0,073	76,83	0,1210
Prevotellaceae	0,007	0,005	0,039	0,028	446,02	0,2954
Pseudomonadaceae	0,003	0,002	0,000	0,000	WT ND	0,0668
Rhodobacteraceae	0,000	0,000	0,001	0,001	*Napepld*$^{\Delta IEC}$ ND	0,3574
Rikenellaceae	11,022	1,064	12,320	0,896	11,77	0,3610
Ruminococcaceae	19,297	1,118	17,210	1,020	-10,81	0,1849
S24-7	6,620	1,025	9,463	0,873	42,93	0,0487
Streptococcaceae	0,004	0,003	0,006	0,003	24,48	0,8020
Turicibacteraceae	0,023	0,016	0,029	0,027	28,89	0,8420
u Bacteroidale	7,275	0,676	8,616	1,361	18,44	0,4061
u Clostridiale	31,657	1,204	27,154	1,511	-14,23	0,0347
u Lactobacillale	0,000	0,000	0,001	0,001	*Napepld*$^{\Delta IEC}$ ND	0,3574
u RF32	0,533	0,101	1,183	0,380	121,98	0,1340
u RF39	0,179	0,031	0,462	0,220	158,80	0,2433
u YS2	0,107	0,030	0,101	0,045	-5,48	0,9162
Unassigned	1,158	0,132	0,986	0,087	-14,83	0,2852

	WT ND		WT HFD			
	Mean	SEM	Mean	SEM	Changes (%)	p-value
Alcaligenaceae	0,439	0,127	0,001	0,001	-99,78	0,0020*
Anaeroplasmataceae	1,299	0,558	0,000	0,000	WT ND	0,0247
Bacteroidaceae	0,922	0,128	1,920	0,172	108,09	0,0003*
Brucellaceae	0,000	0,000	0,001	0,001	WT HFD	0,3574
Caulobacteraceae	0,001	0,001	0,000	0,000	WT ND	0,3051
Christensenellaceae	0,090	0,021	0,021	0,006	-77,10	0,0038*
Clostridiaceae	0,016	0,008	0,010	0,004	-39,38	0,5049
Comamonadaceae	0,000	0,000	0,001	0,001	WT HFD	0,3574
Coriobacteriaceae	0,038	0,019	0,005	0,002	-88,01	0,0799
Deferribacteraceae	4,528	1,056	1,735	0,266	-61,68	0,0154
Dehalobacteriaceae	0,299	0,028	0,250	0,033	-16,15	0,2832
Desulfovibrionaceae	4,926	0,285	7,910	0,307	60,57	0,000002*
Enterobacteriaceae	0,002	0,002	0,003	0,002	49,52	0,7016
Erysipelotrichaceae	2,529	0,774	0,092	0,046	-96,37	0,0040*
Eubacteriaceae	0,001	0,001	0,000	0,000	WT ND	0,3051
F16	0,048	0,022	0,000	0,000	WT ND	0,0309
Lachnospiraceae	2,522	0,298	2,466	0,167	-2,24	0,8673
Lactobacillaceae	0,088	0,029	0,053	0,021	-40,27	0,3247
Microbacteriaceae	0,000	0,000	0,002	0,002	WT HFD	0,3574
Mogibacteriaceae	0,107	0,020	0,082	0,018	-23,42	0,3635
Odoribacteraceae	3,870	0,234	2,649	0,223	-31,55	0,0015*
Peptococcaceae	0,196	0,039	0,582	0,066	197,25	0,0001*
Phyllobacteriaceae	0,000	0,000	0,001	0,001	WT HFD	0,3574
Porphyromonadaceae	0,192	0,050	0,287	0,063	49,72	0,2581
Prevotellaceae	0,007	0,005	0,026	0,014	259,73	0,2403
Pseudomonadaceae	0,003	0,002	0,001	0,001	-65,91	0,3064
Rhodobacteraceae	0,000	0,000	0,001	0,001	WT HFD	0,3574
Rikenellaceae	11,022	1,064	15,613	0,933	41,65	0,0046*
Ruminococcaceae	19,297	1,118	26,504	0,802	37,34	0,0001*
*S24-7	6,620	1,025	4,468	0,326	-32,52	0,0517
Streptococcaceae	0,004	0,003	0,007	0,006	56,31	0,7300
Turicibacteraceae	0,023	0,016	0,000	0,000	WT ND	0,1514
u Bacteroidale	7,275	0,676	5,579	0,372	-23,31	0,0373
u Clostridiale	31,657	1,204	28,646	1,094	-9,51	0,0809
u RF32	0,533	0,101	0,130	0,050	-75,64	0,0018*
u RF39	0,179	0,031	0,103	0,029	-42,46	0,0886
u YS2	0,107	0,030	0,051	0,020	-52,02	0,1302
Unassigned	1,158	0,132	0,802	0,095	-30,76	0,0401

	WT HFD		*Napepld*$^{\Delta IEC}$ HFD			
	Mean	SEM	Mean	SEM	Changes (%)	p-value
Alcaligenaceae	0,001	0,001	0,000	0,000	WT HFD	0,3306
Anaeroplasmataceae	0,000	0,000	0,039	0,039	WT HFD	0,3306
Bacteroidaceae	1,920	0,172	1,857	0,285	-3,27	0,8527
Brucellaceae	0,001	0,001	0,000	0,000	WT HFD	0,3306
Caulobacteraceae	0,000	0,000	0,001	0,001	*Napepld*$^{\Delta IEC}$ HFD	0,3306
Christensenellaceae	0,021	0,006	0,028	0,009	34,56	0,5142
Clostridiaceae	0,010	0,004	0,023	0,020	143,68	0,5119
Comamonadaceae	0,001	0,001	0,000	0,000	WT HFD	0,3306
Coriobacteriaceae	0,005	0,002	0,006	0,004	27,57	0,7712
Deferribacteraceae	1,735	0,266	2,482	0,471	43,04	0,1847
Dehalobacteriaceae	0,250	0,033	0,240	0,028	-4,00	0,8190
Desulfovibrionaceae	7,910	0,307	8,099	0,499	2,40	0,7501
Enterobacteriaceae	0,003	0,002	0,007	0,005	102,23	0,5059
Erysipelotrichaceae	0,092	0,046	0,055	0,023	-40,09	0,4825
F16	0,000	0,000	0,011	0,006	*Napepld*$^{\Delta IEC}$ HFD	0,1029
Lachnospiraceae	2,466	0,167	2,668	0,238	8,21	0,4952
Lactobacillaceae	0,053	0,021	0,061	0,027	16,48	0,8042
Microbacteriaceae	0,002	0,002	0,000	0,000	WT HFD	0,3306
Mogibacteriaceae	0,082	0,018	0,045	0,009	-44,83	0,0879
Odoribacteraceae	2,649	0,223	2,211	0,225	-16,56	0,1830
Peptococcaceae	0,582	0,066	0,530	0,063	-8,87	0,5792
Phyllobacteriaceae	0,001	0,001	0,000	0,000	WT HFD	0,3306
Porphyromonadaceae	0,287	0,063	0,220	0,039	-23,25	0,3795
Prevotellaceae	0,026	0,014	0,006	0,004	-78,04	0,1794
Pseudomonadaceae	0,001	0,001	0,008	0,004	543,88	0,1249
Rikenellaceae	15,613	0,933	14,145	1,152	-9,40	0,3351
Ruminococcaceae	26,504	0,802	24,676	1,313	-6,90	0,2503
S24-7	4,468	0,326	3,667	0,327	-17,92	0,0998
Streptococcaceae	0,007	0,006	0,000	0,000	WT HFD	0,2737
u Bacteroidale	5,579	0,372	4,538	0,485	-18,67	0,1056
u Clostridiale	28,646	1,094	33,442	2,106	16,74	0,0584
u RF32	0,130	0,050	0,060	0,032	-53,92	0,2569
u RF39	0,103	0,029	0,044	0,009	-57,28	0,0681
u YS2	0,051	0,020	0,029	0,018	-42,87	0,4284
Unassigned	0,802	0,095	0,802	0,078	0,02	0,9990

All families detected by pyrosequencing are presented in the tables. Indication WT ND, *Napepld*$^{\Delta IEC}$ ND, WT HFD or *Napepld*$^{\Delta IEC}$ HFD in the column ‘changes (%)’ corresponds to the group of mice for which the given phylotype was found exclusively. P-values are based on Student *t*-test. u=unclassified. Significant P-values (≤0.05) are indicated in red.

*: significant p-values after FDR correction.

Supplementary Table 3 : qPCR primers used in this study.

Gene	Forward primer sequence (5'-3')	Reverse primer sequence (5'-3')
Acaca	TGTTGAGACGCTGGTTTGTAGAA	GGTCCTTATTATTGTCCCAGACGTA
Acadm	GCAGAGAAGAAGGGTGACGA	CGTGCCAACAAGAAATACCA
Acox1	CTATGGGATCAGCCAGAAAGG	AGTCAAAGGCATCCACCAAAG
Agrp	CGGAGGTGCTAGATCCACAGA	AGGACTCGTGCAGCCTTACAC
ApoA4	GCACAACAAGCTGGTGCCC	CAGGTGCTCCTGCAACTTCTG
ApoB	GGAAGCTGTTGCTGAGTGGT	ACCCTTCTCTGATGGCCTGT
ApoE	RRCCGTCATAGTGTCCTCCA	CAGAGCTCCCAAGTCACACA
Cart	TTCCTGCAATTCTTTCCTCTTGA	GGGAATATGGGAACCGAAGGT
Cd11c	ACGTCAGTACAAGGAGATGTTGGA	ATCCTATTGCAGAATGCTTCTTTACC
Cd36	GCCAAGCTATTGCGACATGA	ATCTCAATGTCCGAGACTTTTCAAC
Cpt1a	AGACCGTGAGGAACTCAAACCTAT	TGAAGAGTCGCTCCCACT
Cnr1	CTGATGTTCTGGATCGGAGTC	TCTGAGGTGTGAATGATGATGC
Col6a1	AGGATGGACCACCAGGAAAC	TCTCCCACTTCACCCTCATC
Col6a2	CCTGGACAGAAAGGACGACA	ACCATCCGATCCAAATTCAC
Dgat2	ACTCTGGAGGTTGGCACCAT	GGGTGTGGCTCAGGAGGAT
Dll4	GGAACCTTCTCACTCAACATCC	CTCGTCTGTTCGCCAAATCT
Elovl3	TTCTCACGCGGGTTAAAAATGG	GAGCAACAGATAGACGACCAC
Col6a3	AAGAATGGCGTGAAGTTTGG	ATTGCTGTTGGTTTGGTCGT
Faah	ACAGGCAGGCCTATACCCTT	GAGATGTATCGCCAGTCCGT
Fabp1	GCAGAGCCAGGAGAACTTTG	TGATGTCCTTCCCTTTCTGG
Fasn	TTCCAAGACGAAAATGATGC	AATTGTGGGATCAGGAGAGC
Fatp4	GGCTCTCATCAACACCAACC	CCAGGATCCAGAGCAGAAGA
F4/80	TGACAACCAGACGGCTTGTG	GCAGGCGAGGAAAAGATAGTGT
Flt4	CTGGCCAGAGGCACTAAGAC	CAGGGTGTCCTCTGGGAATA
Gpam	TTATCACCAGGACGGAAAGG	TCTCTTTGAAAACCCCGATG
Gpr119	AGCTCTGCTCAGCATACACAG	AAATGCCATCCGAAGGCTAC
Il1b	TCGCTCAGGGTCACAAGAAA	CATCAGAGGCAAGGAGGAAAAC
Ldlr	TGTGGGCTCCATAGGCTATCT	GCGGTCCAGGGTCATCTTC
Lyve1	TGGTGTTACTCCTGCCCTCT	TTCTGCGCTGACTCTACCTG
Lbp	GTCCTGGGAATCTGTCCTTG	CCGGTAACCTTGCTGTTGTT
Math1	CAAGTGTGTCCAGCAGTGTG	TTGAGTTTCTTCAAGGCGGC
Mcp1	GCAGTTAACGCCCCACTCA	CCCAGCCTACTCATTGGGATCA
Me1	CCGGCTCTATCCTCCTTTG	CAGTGGCCATCTTTTCTTTGT
Mgl	ATGGTCCTGATTTCACCTCTGGT	TCAACCTCCGACTTGTTCCGAGACA
Mttp	ATGATCCTCTTGGCAGTGCTT	TGAGAGGCCAGTTGTGTGAC
Naaa	ATTATGACCATTGGAAGCCTGCA	CGCTCATCACTGTAGTATAAATTGTGTAG
Napepld	GCAAGGTCAAAAGGACCAAA	TTCTTTGCTGGGGATACTGG
Npc1L1	GGCTCCATCTGGAGTAGCTG	ATCGCACTACCATCCAGGAC
Npy	CAGAAAACGCCCCCAGAAC	CGGGAGAACAAGTTTCATTTCC
Oxytocin	CCTGGATATGCGCAAGTGT	GGCGAAGGCAGGTAGTTCTC
Pai1	ACAGCCTTTGTCATCTCAGCC	CCGAACCACAAAGAGAAAGGA
Pgc1a	AGCCGTGACCACTGACAACGAG	GCTGCATGGTTCTGAGTGCTAAG
Pparα	CAACGGCGTCGAAGACAAA	TGACGGTCTCCACGGACAT
Pomc	AGGCCTGACACGTGGAAGAT	AGCAGGAGGGCCAGCAA
Rpl19	GAAGGTCAAAGGGAATGTGTTCA	CCTTGTCTGCCTTCAGCTTGT
Scd1	CCTCTTCGGGATTTTCTACTACATG	GCCGTGCCTTGTAAGTTCTGT
Srebf1	GATCAAAGAGGAGCCAGTGT	TAGATGGTGGCTGCTGAGTG
Tnfa	AGCCCCCAGTCTGTATCCTT	GGTCACTGTCCCAGCATCTT
Ucp1	GCTACACGGGGACCTACAATC	CGTCATCTGCCAGTATTTTGTT

Supplementary Table 4: antibodies used in this study. antibodies used in this study.

Target	Dilution	Reference
Rabbit anti NAPE-PLD	1:200	Ab95397 (Abcam) [1]
Mouse anti β-actin	1:10000	Ab6276 (Abcam) [1]
Rabbit anti-Akt	1:1000	9272S (Cell Signaling) [1]
Rabbit anti p-Akt^{Ser473}	1:1000	4060S (Cell Signaling) [1]
Rabbit anti p-Akt^{Thr308}	1:1000	2965S (Cell Signaling)
Rabbit anti AMPK	1:1000	2603S (cell Signaling) [2]
Rabbit anti p-AMPK^{Thr172}	1:1000	2535S (Cell Signaling) [2]
Mouse anti α-tubulin	1:2000	T6199 (sigma)
Rabbit anti c-Fos	1:2000 (IHC); 1:10000 (IF)	Ab190289 (Abcam) [3]
Rabbit anti-POMC	1:1000	Ab94446 (Abcam)
Rabbit anti-mouse	1:4000	P0260 (Dako)
Goat anti-rabbit, HRP-conjugated	1:10000 (WB)	AP132P (Chemicon)
Goat anti-rabbit, HRP-conjugated	Ready to use (histology)	K4010 (Envision kit, Dako)

Supplementary Table 5: Lysophosphatidylcholines (LPC) and phosphatidylcholines (PC), quantification in the small intestine from different groups (pmol/mg of tissue)

	WT ND		Napepld$^{\Delta IEC}$ ND		WT HFD		Napepld$^{\Delta IEC}$ HFD	
	Mean	SEM	Mean	SEM	Mean	SEM	Mean	SEM
**LPC a C16:0**	186,04	16,82	211,55	26,67	126,44*	7,90	129,79	11,88
**LPC a C18:0**	118,57	7,41	123,23	9,94	123,47	7,30	121,80	7,37
**LPC a C18:1**	33,19	3,24	38,74	4,71	21,17*	2,13	19,64	1,19
**LPC a C18:2**	20,48	1,81	26,09	3,07	19,13	1,67	17,67	0,76
**LPC a C20:4**	7,17	0,73	9,39	1,46	6,50	0,73	7,37	0,47
**LPC e C18:0**	4,57	0,46	5,07	0,44	4,71	0,24	4,75	0,28
**PC aa C30:0**	2,46	0,31	2,19	0,16	1,49*	0,13	1,51	0,22
**PC aa C32:0**	12,34	1,59	11,68	1,37	8,25	0,82	8,96	0,83
**PC aa C32:1**	29,89	2,26	26,39	1,75	8,50*	0,43	9,11	0,97
**PC aa C32:2**	3,22	0,25	3,04	0,23	1,12*	0,08	1,07	0,15
**PC aa C34:0**	3,31	0,49	3,83	0,59	3,17	0,27	3,25	0,42
**PC aa C34:1**	95,06	10,27	86,85	7,01	55,79*	3,82	55,99	5,89
**PC aa C34:2**	694,65	85,01	725,84	80,47	626,68	32,62	597,04	69,16
**PC aa C34:3**	41,96	2,89	42,02	4,94	19,46*	1,20	18,24	2,26
**PC aa C36:0**	4,00	0,45	3,89	0,44	3,90	0,25	3,71	0,35
**PC aa C36:1**	6,60	1,18	4,67	0,66	1,36*	0,55	1,63	0,80
**PC aa C36:2**	171,68	27,97	163,99	19,00	216,93	15,73	198,70	17,19
**PC aa C36:3**	150,37	19,47	138,63	12,43	106,28	8,16	98,08	10,30
**PC aa C36:4**	343,99	30,77	352,53	35,10	237,68*	12,96	279,21	26,01
**PC aa C36:5**	29,32	2,41	29,12	3,58	12,04*	0,65	13,29	1,02
**PC aa C38:1**	0,35	0,06	0,28	0,03	0,19*	0,02	0,23	0,04
**PC aa C38:2**	0,81	0,13	0,63	0,08	0,54	0,10	0,54	0,09
**PC aa C38:3**	8,44	1,42	6,30	0,93	3,52*	0,38	4,13	0,54
**PC aa C38:4**	108,56	12,55	102,88	9,34	97,17	8,33	111,39	9,97
**PC aa C38:5**	65,91	6,48	62,67	4,98	41,33*	2,51	45,17	4,45
**PC aa C38:6**	70,29	6,51	72,32	8,18	50,30*	2,26	55,09	4,71
**PC aa C40:4**	1,26	0,12	1,11	0,17	0,93*	0,09	0,91	0,13
**PC aa C40:5**	2,20	0,24	2,04	0,22	1,16*	0,16	1,44	0,23
**PC aa C40:6**	13,33	1,72	12,39	1,38	11,95	0,94	12,52	0,95
**PC aa C40:7**	7,33	0,75	6,76	0,60	4,47*	0,26	4,77	0,39
**PC aa C40:8**	6,83	0,62	6,81	0,65	6,50	0,40	6,72	0,50
**PC ae C32:0**	1,84	0,23	1,63	0,16	1,04*	0,09	1,06	0,10
**PC ae C32:1**	1,22	0,11	1,08	0,05	0,48*	0,04	0,46	0,07
**PC ae C32:6**	0,02	0,01	0,02	0,01	0,00	0,00	0,01	0,01
**PC ae C34:0**	0,35	0,06	0,40	0,03	0,40	0,05	0,33	0,04
**PC ae C34:1**	6,06	0,66	5,32	0,40	3,53*	0,26	3,88	0,49
**PC ae C34:2**	5,01	0,45	4,39	0,48	3,54*	0,29	3,25	0,48
**PC ae C34:3**	0,62	0,05	0,56	0,06	0,39*	0,04	0,32	0,04
**PC ae C36:1**	2,44	0,22	2,40	0,24	1,84*	0,16	2,06	0,20
**PC ae C36:2**	8,52	1,05	8,98	1,09	10,43	0,88	9,60	0,81
**PC ae C36:3**	2,63	0,28	2,71	0,26	2,28	0,16	2,11	0,23
**PC ae C36:4**	5,84	0,59	5,67	0,38	4,02*	0,27	4,45	0,58
**PC ae C36:5**	3,56	0,36	3,45	0,39	2,32*	0,19	2,43	0,24

**PC ae C38:1**	0,69	0,09	0,63	0,06	0,52	0,03	0,57	0,09
**PC ae C38:2**	2,57	0,53	2,74	0,62	1,91	0,27	1,85	0,22
**PC ae C38:3**	1,55	0,16	1,41	0,11	0,95*	0,08	1,07	0,10
**PC ae C38:4**	5,47	0,53	5,79	0,56	5,17	0,44	5,94	0,57
**PC ae C38:5**	6,91	0,63	7,02	0,42	5,53	0,25	6,48	0,79
**PC ae C38:6**	2,90	0,28	2,84	0,21	1,70*	0,09	1,76	0,22
**PC ae C40:5**	1,01	0,09	0,94	0,08	0,81	0,06	0,82	0,06

*Significantly different from WT ND, P-values <0,05, Student *t*-test.

Supplementary Table 6: Lysophosphatidylethanolamines (LPE) and phosphatidylethanolamines (PE), quantification in the small intestine from different groups (pmol/mg of tissue)

	WT ND		Napepld$^{\Delta IEC}$ ND		WT HFD		Napepld$^{\Delta IEC}$ HFD	
	Mean	SEM	Mean	SEM	Mean	SEM	Mean	SEM
**LPE a C16:0**	1,49	0,10	1,84	0,18	0,95*	0,14	1,10	0,16
**LPE a C18:0**	2,64	0,30	3,20	0,33	2,88	0,56	2,99	0,43
**LPE a C18:1**	0,94	0,07	1,13	0,11	1,09	0,27	0,98	0,11
**LPE a C18:2**	0,42	0,05	0,54	0,03	0,42	0,07	0,42	0,03
**LPE a C20:4**	0,32	0,03	0,44*	0,04	0,25	0,04	0,31	0,01
**LPE a C22:4**	0,06	0,00	0,07	0,00	0,06	0,01	0,06	0,00
**LPE a C22:5**	0,04	0,00	0,05*	0,00	0,04	0,00	0,04	0,00
**LPE a C22:6**	0,23	0,02	0,32*	0,03	0,15*	0,02	0,21$^{\$}$	0,02
**LPE e C18:0**	0,10	0,01	0,12	0,01	0,09	0,01	0,10	0,01
**LPG e C14:2**	0,43	0,08	0,44	0,04	0,45	0,09	0,44	0,05
**PE aa C20:0**	0,03	0,00	0,04	0,01	0,02*	0,00	0,02	0,00
**PE aa C22:2**	0,01	0,00	0,01	0,00	0,01*	0,00	0,01	0,00
**PE aa C26:4**	0,21	0,03	0,22	0,03	0,13*	0,02	0,14	0,01
**PE aa C28:4**	0,05	0,01	0,05	0,01	0,06	0,01	0,06	0,00
**PE aa C28:5**	0,02	0,00	0,02	0,00	0,01	0,00	0,01	0,00
**PE aa C34:0**	0,06	0,01	0,06	0,01	0,03*	0,00	0,04	0,01
**PE aa C34:1**	0,76	0,08	0,77	0,07	0,33*	0,04	0,39	0,05
**PE aa C34:2**	6,34	1,09	6,76	0,78	3,90	0,36	4,34	0,58
**PE aa C34:3**	0,71	0,10	0,65	0,11	0,20*	0,02	0,22	0,03
**PE aa C36:0**	0,38	0,05	0,40	0,04	0,19*	0,02	0,23	0,03
**PE aa C36:1**	1,62	0,20	1,60	0,21	1,04*	0,13	1,19	0,16
**PE aa C36:2**	20,17	3,33	21,06	2,76	19,74	2,13	20,24	2,37
**PE aa C36:3**	8,83	1,55	8,27	1,08	7,44	0,91	7,36	0,98
**PE aa C36:4**	12,33	1,63	13,71	1,17	7,00*	0,71	8,71	0,88
**PE aa C36:5**	1,68	0,19	1,63	0,16	0,51*	0,05	0,62	0,07
**PE aa C38:0**	0,62	0,07	0,64	0,06	0,34*	0,03	0,41	0,04
**PE aa C38:1**	0,23	0,03	0,23	0,02	0,12*	0,01	0,14	0,02
**PE aa C38:2**	0,26	0,04	0,28	0,04	0,16	0,02	0,19	0,02
**PE aa C38:3**	2,32	0,30	2,36	0,28	1,07*	0,11	1,32	0,15
**PE aa C38:4**	27,96	4,15	31,30	3,30	22,66	2,55	28,23	2,36
**PE aa C38:5**	6,87	0,82	7,04	0,55	4,96	0,54	5,89	0,53
**PE aa C38:6**	11,04	1,31	13,06	1,40	6,07*	0,56	8,20	0,90
**PE aa C38:7**	0,72	0,06	0,73	0,06	0,27*	0,02	0,35	0,03
**PE aa C40:2**	0,05	0,01	0,05	0,01	0,03*	0,00	0,03	0,00
**PE aa C40:3**	0,04	0,00	0,04	0,01	0,02*	0,00	0,02	0,00
**PE aa C40:4**	0,64	0,08	0,74	0,10	0,43*	0,05	0,51	0,05
**PE aa C40:5**	0,98	0,12	1,11	0,12	0,62*	0,07	0,74	0,08
**PE aa C40:6**	6,51	0,88	7,38	0,90	3,83*	0,41	4,82	0,47
**PE aa C40:7**	4,09	0,37	4,39	0,42	2,45*	0,24	2,99	0,26
**PE aa C48:1**	0,01	0,00	0,01	0,00	0,01	0,00	0,01	0,00
**PE ae C34:1**	0,11	0,01	0,11	0,01	0,05*	0,00	0,06	0,01
**PE ae C34:2**	0,13	0,01	0,13	0,02	0,07*	0,01	0,08	0,01
**PE ae C34:3**	0,04	0,01	0,04	0,01	0,02*	0,00	0,02	0,00

**PE ae C36:1**	0,25	0,03	0,25	0,03	0,15*	0,02	0,18	0,03
**PE ae C36:2**	1,07	0,12	1,17	0,14	0,82	0,08	0,85	0,09
**PE ae C36:3**	0,28	0,03	0,28	0,03	0,17*	0,01	0,19	0,02
**PE ae C36:4**	0,49	0,04	0,54	0,05	0,27*	0,02	0,32	0,04
**PE ae C36:5**	0,34	0,04	0,35	0,04	0,17*	0,02	0,21	0,03
**PE ae C38:1**	0,17	0,02	0,16	0,03	0,07*	0,01	0,10	0,01
**PE ae C38:2**	0,34	0,05	0,40	0,07	0,20*	0,02	0,23	0,03
**PE ae C38:3**	0,21	0,02	0,22	0,02	0,10*	0,01	0,10	0,01
**PE ae C38:4**	2,74	0,30	3,25	0,36	2,43	0,27	2,86	0,25
**PE ae C38:5**	0,91	0,08	0,98	0,09	0,60*	0,06	0,69	0,08
**PE ae C38:6**	1,61	0,17	1,75	0,18	0,84*	0,08	0,89	0,12
**PE ae C40:1**	1,09	0,11	1,22	0,11	0,65*	0,06	0,80	0,07
**PE ae C40:2**	0,26	0,03	0,30	0,04	0,15*	0,01	0,18	0,02
**PE ae C40:3**	0,08	0,01	0,09	0,01	0,03*	0,00	0,04	0,00
**PE ae C40:4**	1,09	0,15	1,28	0,19	0,85	0,09	1,01	0,10
**PE ae C40:5**	0,73	0,07	0,80	0,09	0,51*	0,05	0,56	0,06
**PE ae C40:6**	2,59	0,31	3,10	0,41	1,58*	0,16	1,85	0,20
**PE ae C42:1**	0,13	0,01	0,13	0,01	0,10	0,01	0,12	0,01
**PE ae C42:2**	0,17	0,02	0,18	0,02	0,10*	0,01	0,12	0,01
**PE ae C46:5**	0,01	0,00	0,01	0,00	0,00*	0,00	0,00	0,00
**PE ae C46:6**	0,11	0,01	0,10	0,01	0,09	0,01	0,09	0,01

*Significantly different from WT ND, P-values <0,05, Student *t*-test. [$]Significantly different from WT HFD, P-values <0,05, Student *t*-test.

Supplementary Table 7: Phosphatidylglycerols (PG), quantification in the small intestine from different groups (pmol/mg of tissue)

	WT ND		Napepld$^{\Delta IEC}$ ND		WT HFD		Napepld$_{\Delta IEC}$ HFD	
	**Mean**	**SEM**	**Mean**	**SEM**	**Mean**	**SEM**	**Mean**	**SEM**
**PG aa C30:0**	0,09	0,01	0,11	0,02	0,04*	0,00	0,09$^{\$}$	0,01
**PG aa C32:0**	0,20	0,04	0,19	0,02	0,10	0,02	0,18$^{\$}$	0,03
**PG aa C32:1**	0,38	0,07	0,30	0,04	0,14*	0,03	0,18	0,02
**PG aa C33:6**	0,04	0,01	0,04	0,01	0,02	0,01	0,03	0,01
**PG aa C34:0**	0,13	0,03	0,13	0,03	0,08	0,03	0,09	0,03
**PG aa C34:1**	9,85	0,86	10,05	0,87	5,54*	0,66	6,63	0,33
**PG aa C34:2**	4,06	0,68	3,79	0,37	3,27	0,45	5,45	0,40
**PG aa C34:3**	0,45	0,07	0,41	0,04	0,20*	0,03	0,35$^{\$}$	0,04
**PG aa C36:0**	0,09	0,01	0,12	0,03	0,08	0,01	0,09	0,02
**PG aa C36:1**	0,72	0,13	0,65	0,07	0,43	0,06	0,63	0,06
**PG aa C36:2**	2,06	0,45	1,70	0,26	2,22	0,33	3,36	0,18
**PG aa C36:3**	0,96	0,17	0,83	0,11	0,85	0,14	1,19	0,12
**PG aa C36:4**	1,18	0,13	1,23	0,14	0,73	0,10	1,15$^{\$}$	0,09
**PG aa C38:5**	0,33	0,05	0,23	0,02	0,19	0,03	0,29	0,04
**PG ae C32:0**	0,05	0,01	0,04	0,01	0,03	0,01	0,05	0,02
**PG ae C34:0**	0,03	0,01	0,04	0,01	0,02	0,00	0,03	0,00
**PG ae C34:1**	0,12	0,02	0,10	0,02	0,06	0,01	0,07	0,01
**PG ae C36:1**	0,24	0,02	0,27	0,04	0,19	0,02	0,25	0,03

*Significantly different from WT ND, P-values <0,05, Student *t*-test. $^{\$}$Significantly different from WT HFD, P-values <0,05, Student *t*-test.

Supplementary Table 8: Phosphatidylserines (PS), quantification in the small intestine from different groups (pmol/mg of tissue)

	WT ND		Napepld$^{\Delta IEC}$ ND		WT HFD		Napepld$^{\Delta IEC}$ HFD	
	Mean	SEM	Mean	SEM	Mean	SEM	Mean	SEM
**PS aa C34:1**	0,15	0,02	0,15	0,01	0,06*	0,01	0,08	0,02
**PS aa C34:2**	0,25	0,03	0,26	0,05	0,20	0,02	0,21	0,03
**PS aa C36:0**	0,07	0,01	0,11	0,01	0,07	0,01	0,07	0,01
**PS aa C36:1**	1,00	0,11	1,02	0,18	0,64*	0,08	0,73	0,08
**PS aa C36:2**	3,96	0,42	4,13	0,43	3,14	0,41	3,03	0,26
**PS aa C36:3**	0,40	0,09	0,33	0,04	0,22	0,03	0,24	0,02
**PS aa C36:4**	0,19	0,02	0,21	0,03	0,15	0,02	0,21	0,03
**PS aa C38:1**	0,02	0,00	0,03	0,01	0,01	0,00	0,02	0,00
**PS aa C38:2**	0,11	0,01	0,12	0,02	0,08	0,01	0,10	0,01
**PS aa C38:3**	0,95	0,10	0,88	0,15	0,47*	0,05	0,58	0,04
**PS aa C38:4**	2,80	0,37	3,04	0,44	2,79	0,40	3,26	0,22
**PS aa C38:5**	0,36	0,04	0,34	0,04	0,26	0,03	0,28	0,03
**PS aa C40:1**	0,01	0,00	0,01	0,00	0,00	0,00	0,01	0,00
**PS aa C40:2**	0,02	0,00	0,02	0,01	0,02	0,00	0,01	0,00
**PS aa C40:3**	0,02	0,01	0,03	0,01	0,01	0,00	0,02	0,00
**PS aa C40:4**	0,51	0,04	0,55	0,08	0,41	0,05	0,41	0,04
**PS aa C40:5**	0,28	0,02	0,31	0,04	0,21	0,04	0,24	0,03
**PS aa C40:6**	1,58	0,19	1,67	0,25	0,98*	0,14	1,06	0,10
**PS aa C40:7**	0,18	0,02	0,17	0,02	0,12	0,01	0,13	0,02
**PS aa C42:1**	0,01	0,00	0,00	0,00	0,00	0,00	0,00$	0,00
**PS aa C42:2**	0,01	0,00	0,01	0,00	0,00	0,00	0,01	0,00
**PS aa C42:4**	0,01	0,00	0,01	0,01	0,01	0,00	0,01	0,00
**PS aa C42:5**	0,02	0,00	0,02	0,01	0,02	0,00	0,01	0,00
**PS ae C34:2**	0,01	0,00	0,01	0,00	0,00	0,00	0,00	0,00
**PS ae C36:1**	0,04	0,01	0,04	0,01	0,03	0,00	0,03	0,00
**PS ae C36:2**	0,08	0,01	0,07	0,01	0,06	0,01	0,06	0,01
**PS ae C38:4**	0,06	0,01	0,06	0,01	0,06	0,01	0,07	0,01

*Significantly different from WT ND, P-values <0,05, Student *t*-test. $Significantly different from WT HFD, P-values <0,05, Student *t*-test.

Supplementary Table 9: sphyngomyelins (SM), quantification in the small intestine from different groups (pmol/mg of tissue)

	WT ND		Napepld$^{\Delta IEC}$ ND		WT HFD		Napepld$^{\Delta IEC}$ HFD	
	**Mean**	**SEM**	**Mean**	**SEM**	**Mean**	**SEM**	**Mean**	**SEM**
**SM C3:0**	0,90	0,06	1,28*	0,12	0,91	0,11	0,76	0,05
**SM C14:0**	3,05	0,53	4,26	0,74	3,98	0,27	3,35	0,20
**SM C15:0**	10,99	2,24	13,25	2,50	12,57	1,00	10,76	0,63
**SM C16:0**	113,03	15,79	105,72	10,72	95,38	6,62	97,05	6,57
**SM C16:1**	5,01	0,65	4,89	0,44	4,22	0,13	4,53	0,29
**SM C17:0**	1,38	0,19	1,40	0,19	1,43	0,11	1,31	0,08
**SM C18:0**	3,94	0,62	3,63	0,53	4,24	0,38	4,35	0,33
**SM C18:1**	1,85	0,19	1,76	0,20	2,09	0,15	1,90	0,13
**SM C19:0**	0,14	0,04	0,26	0,03	0,17	0,06	0,13	0,04
**SM C19:1**	0,14	0,03	0,11	0,01	0,09	0,02	0,09	0,02
**SM C19:2**	0,18	0,01	0,16	0,02	0,13*	0,02	0,14	0,02
**SM C20:1**	0,29	0,21	0,35	0,10	0,44	0,09	0,31	0,10
**SM C20:2**	1,54	0,11	1,54	0,12	0,76*	0,04	0,75	0,08
**SM C21:0**	0,68	0,11	0,78	0,14	0,90	0,17	0,72	0,07
**SM C21:1**	0,33	0,07	0,36	0,08	0,36	0,02	0,33	0,06
**SM C21:2**	0,61	0,07	0,63	0,04	0,43*	0,05	0,51	0,07
**SM C21:3**	0,29	0,05	0,32	0,04	0,21	0,03	0,17	0,05
**SM C22:0**	11,82	1,78	12,93	1,78	12,61	1,02	12,51	1,21
**SM C22:1**	19,33	2,98	17,97	1,74	13,48	0,81	13,03	1,33
**SM C22:2**	26,64	2,88	26,34	3,18	20,90	1,60	23,76	2,11
**SM C22:3**	2,23	0,19	2,09	0,31	0,93*	0,05	1,00	0,15
**SM C23:0**	0,56	0,11	0,57	0,12	0,40	0,07	0,39	0,05
**SM C23:1**	0,57	0,10	0,54	0,06	0,41	0,04	0,47	0,03
**SM C23:2**	0,37	0,07	0,40	0,11	0,34	0,08	0,39	0,06
**SM C23:3**	0,01	0,01	0,05	0,03	0,12*	0,04	0,01	0,01
**SM C24:0**	0,57	0,11	0,53	0,09	0,35	0,05	0,31	0,05
**SM C24:1**	3,86	0,59	4,26	0,59	3,41	0,39	3,59	0,33
**SM C24:2**	9,11	1,18	8,97	0,86	8,25	0,71	9,85	1,23
**SM C24:3**	5,39	0,46	6,22	0,50	3,54*	0,34	4,30	0,41
**SM C24:4**	3,88	0,47	4,29	0,61	3,15	0,23	3,59	0,37
**SM C26:3**	1,06	0,14	0,97	0,10	0,73	0,06	0,77	0,06
**SM C26:4**	0,64	0,13	0,67	0,08	0,68	0,09	0,49	0,09

*Significantly different from WT ND, P-values <0,05, Student *t*-test.

Supplementary Table 10: Ceramides (Cer), quantification in the small intestine from different groups (pmol/mg of tissue)

	WT ND		Napepld$^{\Delta IEC}$ ND		WT HFD		Napepld$^{\Delta IEC}$ HFD	
	**Mean**	**SEM**	**Mean**	**SEM**	**Mean**	**SEM**	**Mean**	**SEM**
**N-C10:0(OH)-Cer**	0,01	0,00	0,01*	0,00	0,01*	0,00	0,01	0,00
**N-C10:0-Cer**	0,32	0,04	0,41	0,04	0,24	0,04	0,27	0,04
**N-C11:0(OH)-Cer**	0,01	0,00	0,02	0,00	0,01*	0,00	0,01	0,00
**N-C12:0(OH)-Cer**	0,01	0,00	0,01	0,00	0,01	0,00	0,01	0,00
**N-C12:0-Cer**	1,63	0,16	2,14	0,25	1,20*	0,11	1,34	0,12
**N-C13:0-Cer(2H)**	0,01	0,00	0,01	0,00	0,01	0,00	0,01	0,00
**N-C14:0(OH)-Cer**	0,01	0,00	0,01	0,00	0,00	0,00	0,00	0,00
**N-C14:0(OH)-Cer(2H)**	0,01	0,00	0,01*	0,00	0,01	0,00	0,01	0,00
**N-C14:0-Cer**	0,01	0,00	0,01	0,00	0,01	0,00	0,01	0,00
**N-C15:0(OH)-Cer**	0,00	0,00	0,00	0,00	0,01	0,00	0,01	0,00
**N-C15:0-Cer**	0,04	0,00	0,04	0,00	0,04	0,00	0,04	0,00
**N-C15:0-Cer(2H)**	0,01	0,00	0,01	0,00	0,01	0,00	0,01	0,00
**N-C16:0(OH)-Cer**	0,03	0,00	0,03	0,00	0,02	0,00	0,02	0,00
**N-C16:0(OH)-Cer(2H)**	0,01	0,00	0,02*	0,00	0,01	0,00	0,01	0,00
**N-C16:0-Cer**	0,43	0,06	0,53	0,08	0,37	0,04	0,42	0,05
**N-C16:0-Cer(2H)**	0,07	0,01	0,06	0,00	0,06	0,01	0,07	0,01
**N-C16:1-Cer**	0,01	0,00	0,01*	0,00	0,00	0,00	0,01	0,00
**N-C16:1-Cer(2H)**	0,01	0,00	0,00	0,00	0,01	0,00	0,00	0,00
**N-C17:0(OH)-Cer**	0,01	0,00	0,01	0,00	0,00	0,00	0,01	0,00
**N-C17:0-Cer**	0,01	0,00	0,01	0,00	0,01	0,00	0,01	0,00
**N-C18:0(OH)-Cer**	0,04	0,01	0,07	0,01	0,03	0,01	0,04	0,01
**N-C18:0(OH)-Cer(2H)**	0,01	0,00	0,01	0,00	0,01	0,00	0,01	0,00
**N-C18:0-Cer**	0,02	0,00	0,03*	0,00	0,02	0,00	0,02	0,00
**N-C18:1-Cer**	0,01	0,00	0,01	0,00	0,01	0,00	0,01	0,00
**N-C19:0(OH)-Cer**	0,01	0,00	0,01	0,00	0,01	0,00	0,00	0,00
**N-C19:0-Cer**	0,01	0,00	0,01	0,00	0,00*	0,00	0,01	0,00
**N-C20:0(OH)-Cer**	0,15	0,05	0,25	0,06	0,19	0,07	0,22	0,03
**N-C20:0(OH)-Cer(2H)**	0,02	0,00	0,02	0,01	0,02	0,01	0,03	0,01
**N-C20:0-Cer**	0,03	0,00	0,03	0,00	0,03	0,00	0,03	0,00
**N-C22:0(OH)-Cer**	0,01	0,00	0,01	0,00	0,01	0,00	0,01	0,00
**N-C22:0-Cer**	0,01	0,00	0,01	0,00	0,00	0,00	0,01	0,00
**N-C24:0(OH)-Cer**	0,01	0,00	0,01	0,00	0,01	0,00	0,01	0,00
**N-C24:0-Cer**	0,01	0,00	0,01	0,00	0,01	0,00	0,01	0,00
**N-C24:0-Cer(2H)**	0,01	0,00	0,01	0,00	0,01	0,00	0,00	0,00
**N-C24:1-Cer**	0,02	0,00	0,02	0,00	0,01	0,00	0,01	0,00
**N-C24:1-Cer(2H)**	0,01	0,00	0,01	0,00	0,01	0,00	0,01	0,00
**N-C25:0-Cer**	0,00	0,00	0,00	0,00	0,01	0,00	0,00*	0,00
**N-C25:0-Cer(2H)**	0,01	0,00	0,01	0,00	0,00*	0,00	0,01	0,00
**N-C26:0(OH)-Cer**	0,00	0,00	0,01	0,00	0,00	0,00	0,00	0,00
**N-C26:0-Cer**	0,01	0,00	0,01	0,00	0,00	0,00	0,00	0,00
**N-C28:0-Cer**	0,00	0,00	0,01	0,00	0,00	0,00	0,00	0,00
**N-C7:0(OH)-Cer**	0,01	0,00	0,01	0,00	0,01	0,00	0,01	0,00
**N-C7:0(OH)-Cer(2H)**	0,00	0,00	0,01*	0,00	0,01*	0,00	0,00	0,00

**N-C7:0-Cer**	0,00	0,00	0,01*	0,00	0,00	0,00	0,00	0,00
**N-C8:0(OH)-Cer**	0,00	0,00	0,01	0,00	0,01	0,00	0,01*	0,00
**N-C8:0(OH)-Cer(2H)**	0,00	0,00	0,00	0,00	0,01	0,00	0,01	0,00
**N-C9:0(OH)-Cer**	0,00	0,00	0,01*	0,00	0,01	0,00	0,01	0,00
**N-C9:0(OH)-Cer(2H)**	0,00	0,00	0,00	0,00	0,01	0,00	0,01	0,00

*Significantly different from WT ND, P-values <0,05, Student *t*-test. $Significantly different from WT HFD, P-values <0,05, Student *t*-test.

Supplementary Table 11. LC-MS conditions for the analysis of NAE e 2-acylglycerols in the jejenum of WT, HFD-WT, HFD-NapepldΔIEC and HFD-NapepldΔIEC plus *A. muciniphila* mice

Name	Ret. Time	MRM	ISTD used
**Linoleoyl-EA**	11,034	324.30 → 62.05	Linoleoyl-EA-D4
**Linoleoyl-EA-D4**	11,007	328.00 → 66.10	
**AEA-D8**	11,113	356.40 → 44.15	AEA-D8
**AEA**	11,171	348.30 → 62.25	
**Palmitoyl-EA**	11,563	300.30 → 62.10	Palmitoyl-EA-D4
**Palmitoyl-EA-D4**	11,543	304.00 → 62.30	
**Oleoyl-EA**	12,162	326.00 → 62.05	Oleoyl-EA-D4
**Oleoyl-EA-D4**	12,136	330.20 → 66.10	
**Stearoyl-EA**	13,183	328.20 →62.10	Stearoyl-EA-D3
**Stearoyl-EA-D3**	13,174	331.10 → 62.20	
**2-Linoleoyl-Glycerol**	11,927	355.20 → 263.30	1-AG-D5
**1-Linoleoyl-Glycerol**	12,11	355.20 → 263.30	1-AG-D5
**1-AG-D5**	12,087	384.50 → 287.20	
**2-AG**	11,984	379.30 → 287.25	1-AG-D5
**1-AG**	12,119	379.30 → 287.25	1-AG-D5
**2-Palmitoyl Glycerol**	12,5	331.10 → 313.25	1-AG-D5

Supplementary References

1. Geurts L*, et al.* Adipose tissue NAPE-PLD controls fat mass development by altering the browning process and gut microbiota. *Nature communications* **6**, 6495 (2015).

2. Qiao A, Jin X, Pang J, Moskophidis D, Mivechi NF. The transcriptional regulator of the chaperone response HSF1 controls hepatic bioenergetics and protein homeostasis. *J Cell Biol* **216**, 723-741 (2017).

3. Guo X, Guo H, Zhao L, Zhang YH, Zhang JX. Two predominant MUPs, OBP3 and MUP13, are male pheromones in rats. *Front Zool* **15**, 6 (2018).
