# Supplementary material for: Intestinal epithelial N-acylphosphatidylethanolamine phospholipase D links dietary fat to metabolic adaptations in obesity and steatosis
Source: Nat Commun. 2019 Jan 28;10:457. doi: 10.1038/s41467-018-08051-7 (PMC6349942; doi:10.1038/s41467-018-08051-7)
Supplement: Supplementary file 2 — Description of Additional Supplementary Files [file 41467_2018_8051_MOESM2_ESM.pdf]

Description of Additional Supplementary Files

File Name: Supplementary Data 1
Description: Relative abundance of the bacterial genera found in the different groups (% of 16S rRNA sequences). Related to Figure 5.
