# Supplementary material for: Intestinal epithelial N-acylphosphatidylethanolamine phospholipase D links dietary fat to metabolic adaptations in obesity and steatosis
Source: Nat Commun. 2019 Jan 28;10:457. doi: 10.1038/s41467-018-08051-7 (PMC6349942; doi:10.1038/s41467-018-08051-7)
Supplement: Supplementary file 4 — Reporting Summary [file 41467_2018_8051_MOESM4_ESM.pdf]

nature research

Corresponding author(s): Patrice D. Cani

Reporting Summary

Nature Research wishes to improve the reproducibility of the work that we publish. This form provides structure for consistency and transparency in reporting. For further information on Nature Research policies, see Authors & Referees and the Editorial Policy Checklist.

Statistical parameters

When statistical analyses are reported, confirm that the following items are present in the relevant location (e.g. figure legend, table legend, main text, or Methods section).

n/a	Confirmed	
☐	☒	The exact sample size (*n*) for each experimental group/condition, given as a discrete number and unit of measurement
☐	☒	An indication of whether measurements were taken from distinct samples or whether the same sample was measured repeatedly
☐	☒	The statistical test(s) used AND whether they are one- or two-sided<br>*Only common tests should be described solely by name; describe more complex techniques in the Methods section.*
☐	☒	A description of all covariates tested
☐	☒	A description of any assumptions or corrections, such as tests of normality and adjustment for multiple comparisons
☐	☒	A full description of the statistics including central tendency (e.g. means) or other basic estimates (e.g. regression coefficient) AND variation (e.g. standard deviation) or associated estimates of uncertainty (e.g. confidence intervals)
☐	☒	For null hypothesis testing, the test statistic (e.g. *F*, *t*, *r*) with confidence intervals, effect sizes, degrees of freedom and *P* value noted<br>*Give P values as exact values whenever suitable.*
☒	☐	For Bayesian analysis, information on the choice of priors and Markov chain Monte Carlo settings
☒	☐	For hierarchical and complex designs, identification of the appropriate level for tests and full reporting of outcomes
☒	☐	Estimates of effect sizes (e.g. Cohen's *d*, Pearson's *r*), indicating how they were calculated
☐	☒	Clearly defined error bars<br>*State explicitly what error bars represent (e.g. SD, SE, CI)*

Our web collection on statistics for biologists may be useful.

Software and code

Policy information about availability of computer code

Data collection	N/A
Data analysis	N/A

For manuscripts utilizing custom algorithms or software that are central to the research but not yet described in published literature, software must be made available to editors/reviewers upon request. We strongly encourage code deposition in a community repository (e.g. GitHub). See the Nature Research guidelines for submitting code & software for further information.

Data

Policy information about availability of data

All manuscripts must include a data availability statement. This statement should provide the following information, where applicable:

- Accession codes, unique identifiers, or web links for publicly available datasets
- A list of figures that have associated raw data
- A description of any restrictions on data availability

All lipidomic data are included in this article as supplementary information (Supplementary tables 6-11). All the remaining data will be made available by the corresponding authors upon reasonable request

Field-specific reporting

Please select the best fit for your research. If you are not sure, read the appropriate sections before making your selection.

☒ Life sciences ☐ Behavioural & social sciences ☐ Ecological, evolutionary & environmental sciences

For a reference copy of the document with all sections, see nature.com/authors/policies/ReportingSummary-flat.pdf

Life sciences study design

All studies must disclose on these points even when the disclosure is negative.

Sample size	Number of mice allocated per group was based on previous experiments investigating diet-induced obesity.
Data exclusions	Exclusion criteria were predefined as follows: Mice displaying abnormal behavior when on a HFD (for example, increased aggressiveness leading to alteration of food intake and/or body weight loss) during the follow-up period were excluded from analyses. All tissues were carefully examined during necropsy and sampling. Any mouse displaying lesions (for example, granulous liver) was also excluded. Finally, for all analyses and for each group, any exclusion decision was supported by the use of the Grubbs test for outlier detection.
Replication	At least three independent cohorts have been used to investigate the phenotype of the mice (body weight, fat mass, glucose metabolism, food intake, liver steatosis).
Randomization	At the beginning of each experiment, cages were randomly assigned to experimental groups to ensure that each group was matched in terms of body weight and fat mass.
Blinding	No blinding procedure was followed.

Reporting for specific materials, systems and methods

Materials & experimental systems

n/a	Involved in the study	
☐	☒	Unique biological materials
☐	☒	Antibodies
☒	☐	Eukaryotic cell lines
☒	☐	Palaeontology
☐	☒	Animals and other organisms
☒	☐	Human research participants

Methods

n/a	Involved in the study	
☒	☐	ChIP-seq
☒	☐	Flow cytometry
☒	☐	MRI-based neuroimaging

Unique biological materials

Policy information about availability of materials

Obtaining unique materials	The knock-out mice are not commercially available and have been generated by the authors.

Antibodies

Antibodies used	Table S5 contains all the names/catalog numbers and dilutions of the antibodies used in this study
Validation	All the antibodies have been previously characterized, a citation is provided in the methods.

Animals and other organisms

Policy information about studies involving animals; ARRIVE guidelines recommended for reporting animal research

Laboratory animals	Male mice C57bl6J of 8 weeks
Wild animals	This study did not involved wild animals.

Field-collected samples	This study did not involve samples collected from the field.
